# Examining the mediating effects of metabolic syndrome components on the relationship between dairy product consumption and nonalcoholic fatty liver disease in Korean adults

**DOI:** 10.1371/journal.pone.0346774

**Published:** 2026-04-10

**Authors:** Hyunyu Jeon, Soo-Hyun Kim, Dayeon Shin

**Affiliations:** 1 Department of Food and Nutrition, Inha University, Incheon, Republic of Korea; 2 Department of Nutritional Science and Food Management, Ewha Womans University, Seoul, Republic of Korea; Sarich Neuroscience Research Institute, AUSTRALIA

## Abstract

**Objective:**

Dairy products are known to improve blood lipid profiles and insulin sensitivity and to reduce risk factors for metabolic syndrome (MetS) and nonalcoholic fatty liver disease (NAFLD). However, the mechanisms through which dairy product consumption influences NAFLD via MetS components remain unclear. This study examined the mediating effects of MetS components on the association between dairy product consumption and NAFLD.

**Methods:**

This study included 12,775 Korean adults from the Korea National Health and Nutrition Examination Survey (KNHANES) 2019–2021. Dairy product intake was assessed using a 24-hour dietary recall. NAFLD was defined using a hepatic steatosis index score >36, and MetS was classified according to the National Cholesterol Education Program Adult Treatment Panel III criteria. Multivariable logistic regression analyses were conducted to examine the associations among dairy intake, NAFLD, and MetS components. Mediation analyses with bootstrapping (n = 1,000) were performed to investigate the mediating effects of individual MetS components on the association between dairy consumption and NAFLD.

**Results:**

Consumption of more than one serving of dairy products was associated with a lower prevalence of NAFLD among women (adjusted odds ratio [AOR], 0.75; 95% confidence interval [CI], 0.59–0.96). Regarding MetS components, intake of one serving of dairy products was associated with lower odds of elevated triglycerides in men (AOR, 0.75; 95% CI, 0.63–0.89). In women, consumption of at least one serving was associated with decreased hyperglycemia (AOR, 0.84; 95% CI, 0.73–0.97), abdominal obesity (AOR, 0.69; 95% CI: 0.55–0.87), low high-density lipoprotein cholesterol (AOR, 0.83; 95% CI: 0.72–0.95), and elevated triglyceride levels (AOR, 0.71; 95% CI, 0.60–0.85). Mediation analyses indicated that, among women, significant proportions of the associations between dairy product consumption and NAFLD were mediated by waist circumference (58.0%), systolic blood pressure (18.2%), and high-density lipoprotein cholesterol (51.7%), whereas no significant mediation effects were observed among men.

**Conclusions:**

Dairy product consumption was associated with a lower prevalence of MetS and NAFLD among women. Mediation analysis further suggested that dairy product consumption may reduce the risk of NAFLD by improving metabolic dysfunction among women.

## Introduction

Nonalcoholic fatty liver disease (NAFLD) is a global health concern, and its worldwide prevalence increased from 25.5% in 2005 to 37.8% in 2016 [1]. In Korea, the prevalence of NAFLD was estimated to reach 30.3% by 2021 [2]. Metabolic syndrome (MetS) is a major contributor to NAFLD, and excess free fatty acids (FFAs) [3], oxidative stress [3], and insulin resistance [4] are well-established risk factors for NAFLD. Insulin resistance promotes the direct influx of glucose into the de novo lipogenesis (DNL) pathway, leading to increased triglyceride synthesis. Consequently, elevated FFAs derived from DNL contribute to hepatic fat accumulation, which plays a central role in the development of NAFLD [5,6].

Dietary factors contribute to the development of NAFLD, including the consumption of dairy products [7,8]. One study reported that individuals in the highest dairy product intake group had a 0.86-fold lower risk of developing NAFLD than those in the lowest intake group [9]. In contrast, a meta-analysis examining food groups and NAFLD reported no significant association between dairy product consumption and NAFLD [10]. Similarly, no association was identified between dairy-related dietary diversity scores and NAFLD [11]. Although dairy product consumption may reduce the risk of NAFLD, existing results remain inconsistent. This inconsistency may be partly because the serving size of dairy products is not standardized internationally, dairy consumption varies by country, and different types of dairy products are consumed.

Previous studies have shown that the nutritional components of dairy products, including whey protein and calcium, may reduce the risk of MetS development. Whey protein supplementation at doses of 0, 5, 10, and 20 g has been shown to decrease postprandial glycemia in a dose-dependent manner in healthy participants [12]. Additionally, dairy proteins act as precursors of angiotensin-I-converting enzyme inhibitory peptides, which have the potential to lower blood pressure [13]. Calcium in dairy products may also confer metabolic benefits by binding bile acids and increasing cholesterol conversion to bile acids when intestinal reabsorption is inhibited [13]. Therefore, both the protein and calcium content in dairy products demonstrate beneficial effects on the components of MetS.

Previous studies have demonstrated that effective management of MetS may reduce the NAFLD risk [14–17]. Dairy products containing whey protein and calcium have been associated with a decreased risk of MetS through various mechanisms, including lowering insulin resistance, controlling blood glucose levels, reducing blood pressure, and improving the lipid profile. However, the relationship between dairy intake and NAFLD remains inconclusive, with several studies showing no inverse association [9–11,18,19]. Furthermore, the specific mechanisms through which dairy consumption affects MetS components and subsequently influences NAFLD risk remain unclear. Therefore, the aim of this study was to investigate the association between dairy product consumption and NAFLD prevalence and to examine whether NAFLD risk can be reduced through dairy consumption and MetS management. Specifically, the objectives of this study were to 1) determine the association between dairy product intake and NAFLD, 2) examine the effects of dairy product consumption on MetS components, 3) evaluate the effects of MetS components on NAFLD, and 4) examine the mediating effect of MetS components on the relationship between dairy product intake and NAFLD among Korean adults.

## Materials and methods

### Dataset and study participants

**Fig 1:**
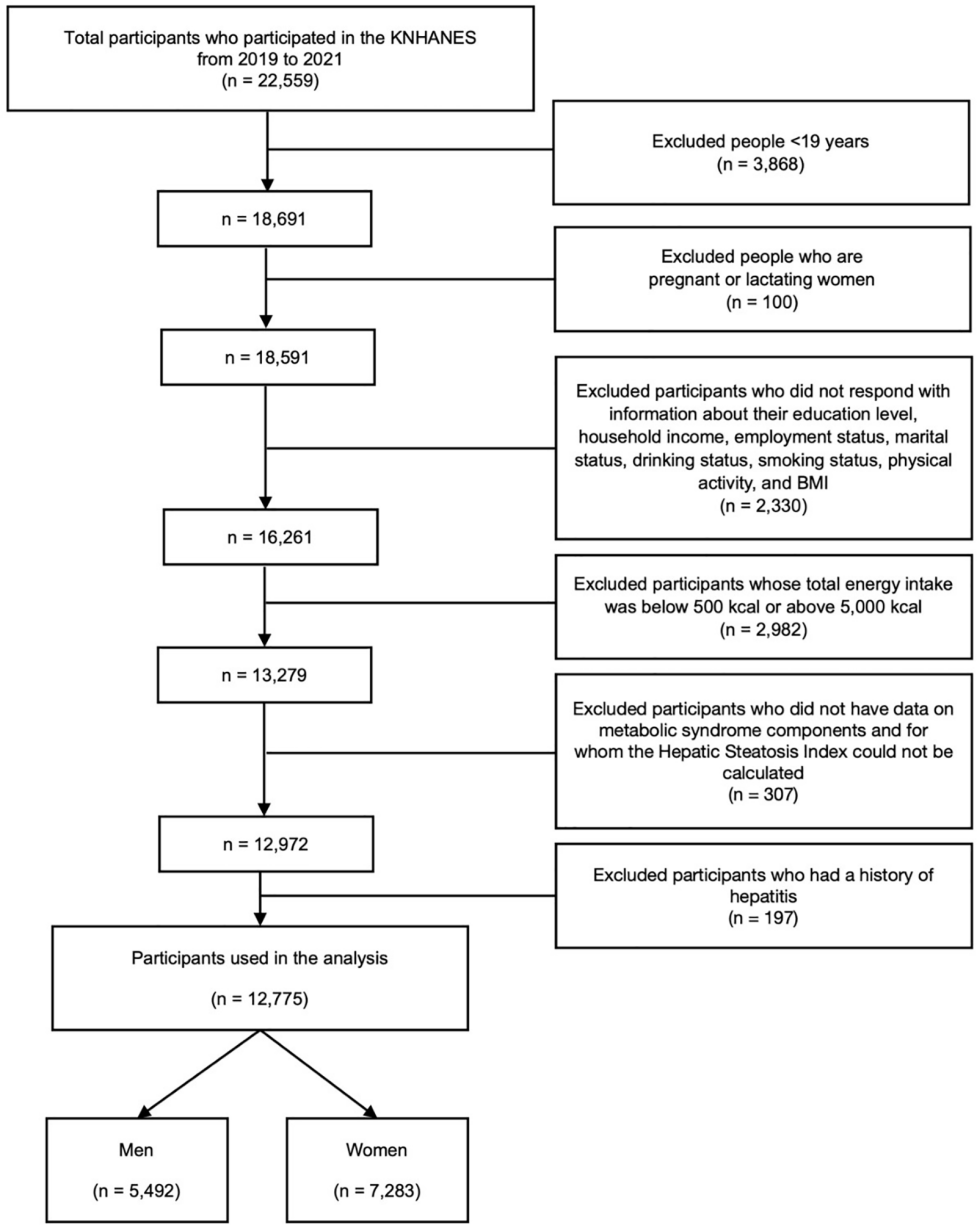
Flow diagram of study participant selection.

This study used data from the Korea National Health and Nutrition Examination Survey (KNHANES) conducted between 2019 and 2021. Among the 22,559 respondents who participated in the KNHANES during this period, individuals aged <19 years (n = 3,868) and those who were pregnant or lactating (n = 100) were excluded. Participants with missing information on covariates, including education level, employment status, household income, marital status, alcohol consumption, smoking status, physical activity, and body mass index (BMI) (n = 2,330), were excluded. Additionally, participants with a total energy intake (<500 kcal or >5,000 kcal; n = 2,982) were excluded. Furthermore, participants who lacked data on MetS components, for whom the hepatic steatosis index (HSI) could not be calculated (n = 307), and those with a history of hepatitis (n = 197) were further excluded. The final sample comprised 12,775 participants (Fig 1). Written informed consent was obtained from all participants before study participation. The dataset was obtained from the Korea Disease Control and Prevention Agency, and the study protocol was approved by the Institutional Review Board (IRB approval no. 2018-01-03-C-A, 2018-01-03-2C-A, and 2018-01-03-5C-A).

### Assessment of dairy product intake

Daily consumption of dairy products was computed from a dietary intake survey using a 1-day 24-hour dietary recall. Dairy products were identified using tertiary food codes and categorized as condensed milk, milk, goat milk, liquid yogurt, semi-solid yogurt, sorbet, ice milk, ice cream, cheese, or cream. These items were converted into serving sizes according to the dietary reference intakes of Koreans [20]. One serving size was defined as 200 g of milk (including condensed milk, milk, and goat milk), 150 g of liquid yogurt, 100 g of semi-solid yogurt, ice cream (including sorbet, ice milk, and ice cream), cream, and 20 g of cheese. Dairy product intake was classified as “0” for no intake, “0–1” for intake of less than one serving, and “≥1” for intake of one or more servings.

### Assessment of nonalcoholic fatty liver disease

The hepatic steatosis index (HSI) is a simple and widely used screening tool for NAFLD. The HSI formula used in this study was as follows: HSI = 8 × (ALT/AST ratio) + BMI + 2 (women) + 2 (diabetes mellitus) [21]. Alanine aminotransferase (ALT) and aspartate aminotransferase (AST) levels were measured using a LaboSpect 008AS (Hitachi, Tokyo, Japan) after fasting for at least 8 h. BMI was calculated as weight (kg) divided by height (m^2^). Height was measured using a Seca 274 stadiometer (Seca, Hamburg, Germany), and weight using a GL-6000–20 scale (G-tech, Seoul, Republic of Korea). Data regarding sex and diabetes status were collected using a self-administered survey. NAFLD was defined using the HSI with a cutoff value of >36.

### Assessment of metabolic syndrome components

According to the National Cholesterol Education Program Adult Treatment Panel III (NCEP ATP III) guidelines, MetS was defined as present when an individual met at least three of the following five criteria: (1) fasting blood glucose ≥100 mg/dL or current use of medication or insulin treatment for hyperglycemia or type 2 diabetes; (2) waist circumference ≥90 cm for men and ≥80 cm for women; (3) systolic blood pressure (SBP) ≥130 mmHg or diastolic blood pressure (DBP) ≥85 mmHg, or current use of antihypertensive medication; (4) HDL cholesterol <40 mg/dL for men and <50 mg/dL for women; and (5) triglycerides ≥150 mg/dL [22]. Blood samples were collected from participants after a fasting period of at least 8 h. HDL cholesterol, triglyceride, and fasting blood glucose levels were measured using a Labospect 008AS (Hitachi, Tokyo, Japan). Waist circumference was assessed at the midpoint between the lowest rib and highest iliac crest using a Lufkin W606 tape measure (Lufkin, USA). SBP and DBP were measured using a non-mercury aneroid sphygmomanometer (WatchBP Office AFIB; Microlife, Switzerland), and the average of these measurements was used.

### Assessment of covariates

Demographic characteristics included sex, age, BMI, education level, household income, employment status, marital status, household type, and region. Sex was classified as “men” or “women,” and education level was categorized as “≤elementary school,” “middle school,” “high school,” or “≥college.” Household income was categorized into quartiles as “low” (quartile 1), “low-medium” (quartile 2), “medium-high” (quartile 3), and “high” (quartile 4). Employment status was classified as “employed” or “unemployed,” and marital status was classified as “single” or “married.” Household type was categorized as “single-person” or “multi-person,” and region was classified as “urban” or “rural.” Health behaviors included alcohol consumption, smoking status, and physical activity. Alcohol consumption was classified as “none” (never or <1 time/month), “moderate” (1–4 times/month), or “high” (more than 2 times/week). Smoking status was classified as “never,” “past,” or “current.” Physical activity was classified as “yes” or “no” based on engagement in at least 2.5 h of moderate-intensity activity, 1.25 h of vigorous-intensity activity, or a combination of both per week.

### Statistical analyses

The complex sampling design of the survey was determined by applying sampling weights, strata, and clusters. For analyses combining multiple survey years, the integrated weight was calculated by multiplying the sampling weight for each year by the integration ratio. To compare general characteristics according to NAFLD status, continuous variables are presented as means and standard errors (SEs) using t-tests and general linear models, and categorical variables are presented as frequencies (n) and percentages (%) using chi-square tests. Multivariable logistic regression was performed to estimate the adjusted odds ratios (AORs) and 95% confidence intervals (CIs) for the association of dairy product intake with NAFLD, dairy product intake with MetS components, and MetS components with NAFLD. To analyze the association between MetS components, each MetS component was categorized into two groups using the same criteria applied in the definition of MetS. All models were adjusted for age, education level, household income, employment status, marital status, household type, region, alcohol consumption, smoking status, total energy intake, physical activity, and BMI. To evaluate the mediating effects of MetS components on the relationship between dairy product consumption and NAFLD, multiple regression analyses were conducted using a bootstrapping method in an SAS macro (n = 1,000). All variables included in the mediation analysis were treated as continuous. MetS components were analyzed as continuous clinical measurements, and NAFLD was represented by a continuous HSI score. Using a continuous HSI score allowed us to capture the full spectrum of hepatic steatosis severity, preserve variability, and satisfy the linearity assumptions of the bootstrapping approach implemented in the SAS macro. All statistical analyses were conducted using SAS software (version 9.4; SAS Institute, Cary, NC, USA). Two-sided *p*-values < 0.05 were considered statistically significant.

## Results

Table 1 presents the general characteristics of the study participants according to the NAFLD prevalence. NAFLD was observed in 29.17% of men and 19.27% of women. The mean age was higher in the NAFLD group than in the control group for both men and women (all *p* < 0.001). BMI was higher in the NAFLD group compared with the normal group in both sexes (all *p* < 0.001). In terms of education level, men were most likely to have college education in both groups, whereas women were most likely to have high school education in the NAFLD group and college education in the normal group (all *p* < 0.001). The most common category of household income was high in both the NAFLD and normal groups among men (*p* < 0.01), whereas among women, household income was medium to high in the NAFLD group and high in the normal group (*p* < 0.001). Employment status showed significant differences according to NAFLD status in both sexes, with a higher proportion of employment in the NAFLD group than in the normal group among men (77.73% vs. 71.54%; *p* < 0.0001), and a higher proportion in the normal group than in the NAFLD group among women (54.73% vs. 51.58%; *p* < 0.05). Alcohol consumption was significantly different according to the presence of NAFLD in both sexes (all *p* < 0.0001), and smoking was significantly different according to the presence of NAFLD only in men (*p* = 0.04). Physical activity was significantly different according to the presence of NAFLD only in women (*p* < 0.01). Consumption of dairy products was not significantly different between the NAFLD group and the normal group in men (*p* = 0.47); meanwhile, for women, dairy product consumption was higher in the normal group (100.03 g/day) than in the NAFLD group (79.01 g/day) (*p* < 0.0001). Consumption of specific dairy products was also not significantly different among men; however, among women, the normal group consumed significantly more milk (*p* < 0.01), semisolid yogurt (*p* = 0.01), and cheese (*p* < 0.001) than the NAFLD group. The prevalence of MetS was significantly higher in the NAFLD group than in the normal group in both sexes (all *p* < 0.001). For clinical measurements of MetS components, the NAFLD group exhibited higher levels of fasting blood glucose, waist circumference, SBP, DBP, and triglycerides and lower levels of HDL cholesterol than the normal group in both men and women (all, *p* < 0.001).

**Table 1:** General characteristics of the study participants with the presence of non-alcoholic fatty liver disease (NAFLD).

Characteristics	Men (n = 5,492)		*p*-value	Women (n = 7,283)		*p*-value
	NAFLD	Normal		NAFLD	Normal	
**Participants (n, %)**	1,450 (29.17%)	4,042 (70.83%)		1,525 (19.27%)	5,758 (80.73%)	
**Age (years)**	48.72 ± 0.38	43.03 ± 0.40	<0.001	52.05 ± 0.51	47.58 ± 0.31	<0.001
**BMI (kg/m**^**2**^)	28.21 ± 0.10	23.35 ± 0.05	<0.001	28.67 ± 0.13	22.09 ± 0.05	<0.001
**Education level (n, %)**			<0.001			<0.001
≤Elementary school	108 (4.37%)	612 (9.55%)		462 (21.79%)	1,172 (14.09%)	
Middle school	90 (4.55%)	439 (8.00%)		189 (11.03%)	534 (7.42%)	
High school	544 (38.37%)	1,423 (37.98%)		498 (38.08%)	1,790 (33.66%)	
≥College	708 (52.71%)	1,568 (44.47%)		376 (29.09%)	2,262 (44.83%)	
**Household income (n, %)**			<0.01			<0.001
Low	179 (9.92%)	777 (13.98%)		355 (17.92%)	1,063 (13.84%)	
Low-medium	325 (21.46%)	990 (22.40%)		429 (27.10%)	1,361 (22.38%)	
Medium-high	436 (30.79%)	1,064 (28.67%)		396 (28.43%)	1,571 (29.49%)	
High	510 (37.84%)	1,211 (34.95%)		345 (26.55%)	1,763 (34.29%)	
**Employment status (n, %)**			<0.001			0.05
Employed	1,105 (77.73%)	2,728 (71.54%)		775 (51.58%)	3,064 (54.73%)	
Unemployed	345 (22.27%)	1,314 (28.46%)		750 (48.42%)	2,694 (45.27%)	
**Marital status (n, %)**			<0.001			<0.001
Single	405 (34.14%)	852(28.66%)		143 (13.81%)	931 (22.55%)	
Married	1,045 (65.86%)	3,190 (71.34%)		1,382 (86.19%)	4,827 (77.45%)	
**Household type (n, %)**			0.76			0.83
Single-person households	194 (12.62%)	564 (12.25%)		234 (11.02%)	845 (10.83%)	
Multi-person households	1,256 (87.38%)	3,478 (87.75%)		1,291 (88.98%)	4,913 (89.17%)	
**Region (n, %)**			0.14			<0.0001
Urban	1,166 (86.39%)	3,174 (84.58%)		1,155 (82.44%)	4,688 (87.13%)	
Rural	284 (13.61%)	868 (15.42%)		370 (17.56%)	1,070 (12.87%)	
**Alcohol consumption (n, %)**			<0.001			<0.001
None	500 (33.66%)	1,349 (31.46%)		1,026 (64.95%)	3,436 (55.04%)	
Moderate	565 (40.29%)	1,354 (36.16%)		377 (26.48%)	1,699 (33.19%)	
High	385 (26.06%)	1,339 (32.38%)		122 (8.57%)	623 (11.77%)	
**Smoking status (n, %)**			0.04			0.28
Never	381 (27.80%)	1,031 (27.71%)		1,329 (85.70%)	5,117 (87.57%)	
Past	597 (38.36%)	1,845 (42.13%)		106 (7.54%)	374 (6.92%)	
Current	472 (33.84%)	1,166 (30.15%)		90 (6.76%)	267 (5.50%)	
**Physical activity (n, %)**			0.09			<0.01
Yes	799 (53.63%)	2,171 (50.76%)		979 (61.76%)	3,390 (56.35%)	
No	651 (46.37%)	1,871 (49.24%)		546 (38.24%)	2,368 (43.65%)	
**Dairy products (g/day)**	79.89 ± 4.41	76.20 ± 2.85	0.47	79.01 ± 4.86	100.03 ± 2.68	<0.001
**Types of dairy products (g/day)**						
Milk	54.80 ± 3.93	52.05 ± 2.36	0.56	54.98 ± 4.37	69.45 ± 2.32	<0.01
Liquid yogurt	3.34 ± 0.82	3.10 ± 0.41	0.79	3.04 ± 0.55	3.23 ± 0.30	0.77
Semisolid yogurt	10.93 ± 1.57	11.15 ± 0.92	0.90	12.44 ± 1.37	16.53 ± 0.93	0.01
Ice cream	8.36 ± 1.00	7.61 ± 0.66	0.52	6.68 ± 1.16	7.64 ± 0.54	0.43
Cheese	1.78 ± 0.34	1.79 ± 0.19	0.97	1.02 ± 0.18	2.21 ± 0.17	<0.001
Cream	0.69 ± 0.19	0.51 ± 0.16	0.47	0.84 ± 0.27	0.96 ± 0.14	0.70
**MetS (n, %)**			<0.001			<0.001
Yes	908 (59.20%)	1,083 (22.71%)		1,062 (66.59%)	1,295 (17.36%)	
No	542 (40.80%)	2,959 (77.29%)		463 (33.41%)	4,463 (82.64%)	
**Fasting blood glucose (mg/dL)**	108.33 ± 0.89	100.61 ± 0.37	<0.001	110.05 ± 0.87	95.13 ± 0.24	<0.001
**Waist circumference (cm)**	96.50 ± 0.26	84.49 ± 0.15	<0.001	92.81 ± 0.29	76.56 ± 0.14	<0.001
**Blood pressure (mmHg)**						
SBP	123.06 ± 0.40	119.33 ± 0.29	<0.001	122.97 ± 0.47	113.91 ± 0.30	<0.001
DBP	80.82 ± 0.31	76.20 ± 0.20	<0.001	76.89 ± 0.31	72.29 ± 0.18	<0.001
**HDL cholesterol (mg/dL)**	44.18 ± 0.30	49.92 ± 0.23	<0.001	49.48 ± 0.30	57.99 ± 0.22	<0.001
**Triglycerides (mg/dL)**	188.75 ± 4.08	135.50 ± 2.11	<0.001	143.17 ± 2.46	98.43 ± 0.93	<0.001

Categorical variables are presented as numbers (%), and continuous variables are presented as means ± standard errors (SEs).

The *p-*values were estimated using the t-test for continuous variables and the chi-square test for categorical variables.

NAFLD was defined as an HSI > 36.

Abbreviations: NAFLD, nonalcoholic fatty liver disease; HSI, hepatic steatosis index; BMI, body mass index; MetS, metabolic syndrome; SBP, systolic blood pressure; DBP, diastolic blood pressure; HDL, high-density lipoprotein.

Nutrient intake of the participants according to their NAFLD status is shown in Table 2. The total energy intake was not significantly different between the NAFLD and normal groups in either sex. Regarding the macronutrient energy distribution, the percentage of energy from carbohydrates was higher in the NAFLD group (62.15%) than in the normal group (60.30%) among women (*p* < 0.0001), whereas no significant differences were observed in the percentage of energy from protein across groups in both sexes. Among men, fat intake percentage was higher in the NAFLD group than in the normal group (22.90% vs. 21.92%; *p* = 0.002), whereas among women, fat intake was higher in the normal group than in the NAFLD group (22.96% vs. 21.39%; *p* < 0.0001). Men in the NAFLD group consumed more fat (58.37 g/day; *p* = 0.02) than women consumed in the normal group (42.69 g/day; *p* = 0.003). The intake of saturated, monounsaturated, and polyunsaturated fatty acid total fat was higher in the NAFLD group than in the normal group in men (*p* = 0.04, *p* = 0.02, *p* = 0.04, respectively), whereas it was higher in the normal group than in the NAFLD group in women (*p* = 0.003, *p* = 0.01, and *p* = 0.003, respectively). For micronutrients, dairy vitamin E, vitamin C, and calcium intake were significantly lower in women with NAFLD (*p* = 0.03, *p* = 0.02, *p* = 0.04, respectively), whereas no significant differences were observed among men. Sodium intake was higher in men in the NAFLD group than in those in the normal group (*p* = 0.03), whereas no significant difference was observed in women.

**Table 2:** Nutrient intake of the participants by the status of nonalcoholic fatty liver disease (NAFLD) status.

Variables	Men (n = 5,492)		*p*-value	Women (n = 7,283)		*p*-value
	NAFLD	Normal		NAFLD	Normal	
Total energy intake (kcal)	2,216.14 ± 24.98	2,171.38 ± 16.25	0.13	1,589.03 ± 22.08	1,610.99 ± 10.68	0.36
% energy of carbohydrate	56.89 ± 0.43	57.67 ± 0.27	0.11	62.15 ± 0.41	60.30 ± 0.21	<0.0001
% energy of protein	15.38 ± 0.16	15.18 ± 0.09	0.25	15.03 ± 0.15	14.99 ± 0.08	0.84
% energy of fat	22.90 ± 0.28	21.92 ± 0.19	0.002	21.39 ± 0.30	22.96 ± 0.17	<0.0001
Carbohydrate (g/day)	304.25 ± 3.22	301.34 ± 2.14	0.44	241.66 ± 3.12	237.49 ± 1.59	0.23
Protein (g/day)	84.92 ± 1.21	82.43 ± 0.80	0.08	59.74 ± 1.13	60.39 ± 0.50	0.58
Fat (g/day)	58.37 ± 1.18	55.18 ± 0.75	0.02	39.50 ± 0.97	42.69 ± 0.48	0.003
Saturated fatty acid (g/day)	18.43 ± 0.41	17.48 ± 0.26	0.04	12.54 ± 0.36	13.69 ± 0.18	0.003
Monounsaturated fatty acid (g/day)	19.46 ± 0.47	18.18 ± 0.30	0.02	12.69 ± 0.35	13.65 ± 0.17	0.01
Polyunsaturated fatty acids (g/day)	14.62 ± 0.31	13.89 ± 0.19	0.04	10.05 ± 0.24	10.84 ± 0.13	0.003
Dietary fiber (g/day)	27.38 ± 0.37	28.18 ± 0.26	0.07	23.89 ± 0.40	23.24 ± 0.20	0.14
Vitamin A (μg RAE/day)	423.66 ± 14.24	414.88 ± 8.65	0.60	358.73 ± 9.90	379.58 ± 5.89	0.06
Vitamin E (mg α-TE/day)	7.90 ± 0.12	7.77 ± 0.08	0.35	6.02 ± 0.10	6.28 ± 0.06	0.03
Vitamin C (mg/day)	74.69 ± 6.02	65.32 ± 1.69	0.12	60.55 ± 2.14	66.24 ± 1.29	0.02
β-Carotene (μg/day)	2,973.29 ± 77.17	3,093.81 ± 72.35	0.26	2,732.49 ± 85.61	2,730.28 ± 46.22	0.98
Magnesium (mg/day)	331.53 ± 4.02	339.58 ± 2.65	0.10	270.33 ± 4.19	269.89 ± 1.99	0.92
Zinc (mg/day)	11.76 ± 0.16	11.72 ± 0.11	0.85	8.70 ± 0.14	8.71 ± 0.06	0.93
Calcium (mg/day)	532.29 ± 8.50	534.26 ± 5.50	0.84	441.22 ± 8.76	460.37 ± 4.36	0.04
Phosphorus (mg/day)	1,196.37 ± 13.75	1,198.31 ± 9.55	0.91	915.38 ± 14.44	931.78 ± 6.67	0.27
Sodium (mg/day)	3,958.56 ± 61.80	3,803.15 ± 36.49	0.03	2,772.08 ± .53.79	2,726.20 ± 25.46	0.44
Potassium (mg/day)	2,945.66 ± 34.51	2,999.71 ± 25.77	0.21	2,492.41 ± 40.64	2,478.52 ± 19.02	0.74

Values are presented as mean ± standard error (SE).

The *p-*values were estimated using the t-test for continuous variables.

NAFLD was defined as an HSI > 36.

Abbreviations: NAFLD, nonalcoholic fatty liver disease; HSI, hepatic steatosis index.

The association between dairy product intake and NAFLD is presented in Table 3. Women who consumed at least one serving size of dairy products showed a significant inverse association with NAFLD compared with women who never consumed dairy products (AOR, 0.75; 95% CI, 0.59–0.96), whereas no significant association was observed in men. However, when dairy product intake was measured in grams and according to the type of dairy product, no significant association was observed in either sex.

**Table 3:** Association between dairy product intake and nonalcoholic fatty liver disease (NAFLD).

Men (n = 5,492)	AOR (95% CI)	*p*-value	Women (n = 7,283)	AOR (95% CI)	*p*-value
**Dairy products (serving size)**			**Dairy products (serving size)**		
0 (n = 3,506)	1.00 (Ref.)		0 (n = 3,953)	1.00 (Ref.)	
0 to 1 (n = 656)	1.07 (0.79–1.46)	0.66	0 to 1 (n = 1,113)	0.88 (0.66–1.17)	0.39
≥ 1 (n = 1,330)	1.02 (0.81–1.28)	0.89	≥ 1 (n = 2,217)	0.75 (0.59–0.96)	0.02
**Men (n = 5,492)**	**β ± SE**	**p-value**	**Women (n = 7,283)**	**β ± SE**	**p-value**
Dairy products (g/day)	−0.0001 ± 0.0003	0.7991	Dairy products (g/day)	−0.0001 ± 0.0002	0.6820
Types of dairy products			Types of dairy products		
Milk (g/day)	−0.0001 ± 0.0004	0.7722	Milk (g/day)	−0.0001 ± 0.0002	0.6259
Liquid yogurt (g/day)	−0.0008 ± 0.0016	0.6095	Liquid yogurt (g/day)	0.0004 ± 0.0012	0.7584
Semisolid yogurt (g/day)	0.0000 ± 0.0008	0.9570	Semisolid yogurt (g/day)	0.0001 ± 0.0005	0.8033
Ice cream (g/day)	0.0003 ± 0.0012	0.7827	Ice cream (g/day)	−0.0004 ± 0.0012	0.7417
Cheese (g/day)	0.0010 ± 0.0071	0.8884	Cheese (g/day)	−0.0007 ± 0.0029	0.8096
Cream (g/day)	0.0003 ± 0.0071	0.9667	Cream (g/day)	0.0004 ± 0.0035	0.9086

One serving size of dairy products was defined as 200g for milk, 150g for liquid yogurt, 100 g for semisolid yogurt, ice cream, and cream, and 20 g for cheese. Dairy product intake was classified as “0” for no intake, “0–1” for consumption exceeding 0 g and intake of less than one serving size, and “≥1” for intake of more than one serving size.

Multivariable logistic regression and linear regression were used to estimate the associations between dairy product intake and components of metabolic syndrome, adjusting for age, BMI, education level, occupation, marital status, household income, household type, region, drinking, smoking, total energy intake, and physical activity.

Abbreviations: NAFLD, non-alcoholic fatty liver disease; AOR, adjusted odds ratio; CI, confidence interval; SE, standard error.

Table 4 shows the association between dairy product intake and MetS components. Among men, consumption of at least one serving of dairy products was associated with significantly lower odds of elevated triglycerides (AOR, 0.75; 95% CI, 0.63–0.89) compared with no dairy consumption. Among women, consumption of 0–1 serving of dairy products was associated with a significantly lower odds of low HDL cholesterol (AOR, 0.79; 95% CI, 0.65–0.96) compared with no dairy consumption. Additionally, women consuming at least one serving of dairy products had significantly lower odds of hyperglycemia (AOR, 0.84; 95% CI, 0.73–0.97), abdominal obesity (AOR, 0.69; 95% CI, 0.55–0.87), low HDL cholesterol (AOR, 0.83; 95% CI, 0.72–0.95), and elevated triglycerides (AOR, 0.71; 95% CI, 0.60–0.85), compared with women who never consumed dairy products.

**Table 4:** Association between dairy product intake and components of metabolic syndrome.

	Hyperglycemia		Abdominal obesity		Elevated blood pressure		Low HDL-cholesterol			Elevated triglycerides		
Men (n = 5,492)	AOR (95% CI)	*p*-value	AOR (95% CI)	*p*-value	AOR (95% CI)	*p*-value	AOR (95% CI)	*p*-value		AOR (95% CI)		*p*-value
**Dairy products (serving size)**												
0 (n = 3,506)	1.00 (Ref.)		1.00 (Ref.)		1.00 (Ref.)		1.00 (Ref.)			1.00 (Ref.)		
0 to 1 (n = 656)	1.04 (0.83–1.30)	0.72	0.94 (0.69–1.28)	0.68	0.95 (0.76–1.19)	0.65	1.08 (0.84–1.39)	0.53		0.98 (0.79–1.20)		0.81
≥ 1 (n = 1,330)	0.88 (0.74–1.03)	0.11	0.90 (0.71–1.15)	0.40	0.83 (0.69–1.01)	0.06	0.93 (0.78–1.11)	0.40		0.75 (0.63–0.89)		0.001
**Women (n = 7,283)**	**AOR (95% CI)**	***p*-value**	**AOR (95% CI)**	***p*-value**	**AOR (95% CI)**	***p*-value**	**AOR (95% CI)**	***p*-value**		**AOR (95% CI)**		***p*-value**
**Dairy products (serving size)**												
0 (n = 3,953)	1.00 (Ref.)		1.00 (Ref.)		1.00 (Ref.)		1.00 (Ref.)			1.00 (Ref.)		
0 to 1 (n = 1,113)	0.83 (0.69–1.01)	0.06	0.86 (0.66–1.13)	0.27	0.92 (0.75–1.14)	0.43	0.79 (0.65–0.96)	0.02		0.84 (0.69–1.01)		0.07
≥ 1 (n = 2,217)	0.84 (0.73–0.97)	0.02	0.69 (0.55–0.87)	0.002	0.96 (0.81–1.13)	0.60	0.83 (0.72–0.95)	0.01		0.71 (0.60–0.85)		0.0001
	**Fasting blood glucose (mg/dL)**		**Waist circumference (cm)**		**SBP (mmHg)**		**DBP (mmHg)**		**HDL cholesterol (mg/dL)**		**Triglycerides (mg/dL)**	
**Men (n = 5,492)**	**β ± SE**	***p*-value**	**β ± SE**	***p*-value**	**β ± SE**	***p*-value**	**β ± SE**	***p*-value**	**β ± SE**	***p*-value**	**β ± SE**	***p*-value**
Dairy products (g/day)	−0.002 ± 0.002	0.49	−0.0009 ± 0.0004	0.02	−0.003 ± 0.001	0.02	−0.002 ± 0.001	0.02	0.0005 ± 0.001	0.68	−0.037 ± 0.010	0.0003
Milk (g/day)	−0.002 ± 0.003	0.42	−0.001 ± 0.0005	0.22	−0.002 ± 0.001	0.22	−0.003 ± 0.001	0.02	0.001 ± 0.001	0.59	−0.040 ± 0.010	0.0001
Liquid yogurt (g/day)	−0.025 ± 0.009	0.004	−0.006 ± 0.002	0.01	−0.020 ± 0.008	0.01	0.002 ± 0.009	0.83	−0.001 ± 0.006	0.83	0.092 ± 0.098	0.35
Semisolid yogurt (g/day)	0.0005 ± 0.006	0.93	−0.002 ± 0.001	0.05	−0.003 ± 0.004	0.42	0.001 ± 0.003	0.76	0.001 ± 0.003	0.73	−0.057 ± 0.032	0.07
Ice cream (g/day)	0.016 ± 0.014	0.23	−0.001 ± 0.002	0.54	−0.017 ± 0.005	0.002	−0.007 ± 0.004	0.11	−0.004 ± 0.005	0.34	−0.008 ± 0.046	0.86
Cheese(g/day)	−0.035 ± 0.018	0.05	−0.006 ± 0.005	0.27	0.011 ± 0.014	0.46	−0.012 ± 0.015	0.43	−0.001 ± 0.014	0.93	−0.253 ± 0.135	0.06
Cream (g/day)	−0.052 ± 0.014	0.0001	−0.002 ± 0.006	0.72	0.024 ± 0.020	0.23	0.019 ± 0.014	0.16	0.034 ± 0.020	0.10	−0.110 ± 0.186	0.56
	**Fasting blood glucose (mg/dL)**		**Waist circumference (cm)**		**SBP (mmHg)**		**DBP (mmHg)**		**HDL cholesterol (mg/dL)**		**Triglycerides (mg/dL)**	
**Women (n = 7,283)**	**β ± SE**	***p*-value**	**β ± SE**	***p*-value**	**β ± SE**	***p*-value**	**β ± SE**	***p*-value**	**β ± SE**	***p*-value**	**β ± SE**	***p*-value**
Dairy products (g/day)	−0.002 ± 0.002	0.16	−0.001 ± 0.0004	0.03	−0.004 ± 0.001	0.01	−0.001 ± 0.001	0.23	0.003 ± 0.001	0.01	−0.025 ± 0.005	<0.0001
Milk (g/day)	−0.002 ± 0.002	0.31	−0.001 ± 0.0004	0.17	−0.003 ± 0.002	0.04	−0.0005 ± 0.001	0.63	0.003 ± 0.001	0.04	-0.025 ± 0.006	<0.0001
Liquid yogurt (g/day)	-0.011 ± 0.009	0.21	−0.002 ± 0.002	0.29	−0.005 ± 0.010	0.62	0.002 ± 0.007	0.84	0.005 ± 0.009	0.59	0.088 ± 0.049	0.08
Semisolid yogurt (g/day)	−0.003 ± 0.004	0.47	−0.002 ± 0.001	0.02	−0.005 ± 0.003	0.08	−0.003 ± 0.002	0.22	0.004 ± 0.003	0.22	−0.038 ± 0.014	0.01
Ice cream (g/day)	−0.002 ± 0.005	0.72	−0.0001 ± 0.002	0.97	−0.002 ± 0.005	0.70	−0.009 ± 0.004	0.02	−0.0004 ± 0.005	0.93	−0.015 ± 0.023	0.52
Cheese (g/day)	−0.014 ± 0.015	0.35	−0.010 ± 0.005	0.07	−0.007 ± 0.017	0.67	0.010 ± 0.013	0.44	0.074 ± 0.019	0.0001	−0.057 ± 0.065	0.38
Cream (g/day)	0.005 ± 0.016	0.75	0.009 ± 0.006	0.12	−0.018 ± 0.015	0.22	0.004 ± 0.011	0.69	−0.013 ± 0.023	0.57	−0.018 ± 0.069	0.80

One serving size of dairy products was defined as 200g for milk, 150 g for liquid yogurt, 100 g for semisolid yogurt, ice cream, and cream, and 20 g for cheese. Dairy product intake was classified “0 serving size” for 0 intake, “0–1 serving size” for consumption exceeding 0 g and less than one serving size, and “≥1 serving size” for intake of more than one serving size.

Multivariable logistic regression and linear regression were used to estimate the associations between dairy product intake and components of metabolic syndrome, adjusting for age, BMI, education level, occupation, marital status, household income, household type, region, drinking, smoking, total energy intake, and physical activity.

Abbreviations: NAFLD, non-alcoholic fatty liver disease; AOR, adjusted odds ratio; CI, confidence interval; HDL, high-density lipoprotein; SE, standard error.

For every 1 g increase in dairy product intake, waist circumference decreased in both men and women (men: β = −0.0009; women: β = −0.001; all *p* < 0.05). Blood pressure also decreased with increasing dairy intake among men, with significantly lower values observed for both SBP (β = −0.003) and DBP (β = −0.002); among women, a decrease was observed for SBP (β = −0.004). Regarding lipid profiles, HDL cholesterol increased by 0.003 mg/dL among women (*p* = 0.01), whereas triglyceride levels decreased by 0.037 mg/dL among men (*p* = 0.0003) and 0.025 mg/dL among women (*p* < 0.0001).

Table 5 presents the AORs and 95% CIs for NAFLD according to MetS components. All components were significantly associated with NAFLD prevalence in both sexes: hyperglycemia (men: 1.99 [1.60–2.48]; women: 2.82 [2.26–3.52]), abdominal obesity (men: 1.90 [1.43–2.52]; women: 1.47 [1.05–2.06]), elevated blood pressure (men: 1.36 [1.11–1.68]; women: 1.68 [1.32–2.14]), low HDL-cholesterol (men: 1.64 [1.32–2.04]; women: 1.97 [1.61–2.41]), and elevated triglycerides (men: 1.97 [1.61–2.42]; women: 2.19 [1.75–2.73]).

**Table 5:** Adjusted odds ratio (AOR) and 95% confidence intervals (CIs) for nonalcoholic fatty liver disease (NAFLD) according to components of metabolic syndrome.

Men (n = 5,492)	AOR (95% CI)	*p*-value	Women (n = 7,283)	AOR (95% CI)	*p*-value
**Hyperglycemia (mg/dL)**					
< 100 (n = 2,908)	1.00 (Ref.)		< 100 (n = 4,721)	1.00 (Ref.)	
≥ 100 (n = 2,584)	1.99 (1.60–2.48)	<0.0001	≥ 100 (n = 2,562)	2.82 (2.26–3.52)	<0.0001
**Abdominal obesity (cm)**					
< 90 (n = 3,213)	1.00 (Ref.)		< 80 (n = 3,576)	1.00 (Ref.)	
≥ 90 (n = 2,279)	1.90 (1.43–2.52)	<0.0001	≥ 80 (n = 3,707)	1.47 (1.05–2.06)	0.02
**Elevated blood pressure (mmHg)**					
SBP < 130 and DBP < 85 (n = 2,920)	1.00 (Ref.)		SBP < 130 and DBP < 85 (n = 4,484)	1.00 (Ref.)	
SBP ≥ 130 or DBP ≥ 85 (n = 2,572)	1.36 (1.11–1.68)	0.0039	SBP ≥ 130 or DBP ≥ 85 (n = 2,799)	1.68 (1.32–2.14)	<0.0001
**Low HDL-cholesterol (mg/dL)**					
> 40 (n = 4,199)	1.00 (Ref.)		> 50 (n = 4,783)	1.00 (Ref.)	
≤ 40 (n = 1,293)	1.64 (1.32–2.04)	<0.0001	≤ 50 (n = 2,500)	1.97 (1.61–2.41)	<0.0001
**Elevated triglycerides (mg/dL)**					
< 150 (n = 3,555)	1.00 (Ref.)		< 150 (n = 5,839)	1.00 (Ref.)	
≥ 150 (n = 1,937)	1.97 (1.61–2.42)	<0.0001	≥ 150 (n = 1,444)	2.19 (1.75–2.73)	<0.0001

Multivariable logistic regression was used to estimate adjusted odds ratios (AORs) and 95% confidence intervals (CIs), adjusting for age, BMI, education level, occupation, marital status, household income, household type, region, drinking, smoking, total energy intake, and physical activity.

Abbreviations: NAFLD, nonalcoholic fatty liver disease; AOR, adjusted odds ratio; CI, confidence interval; HDL, high-density lipoprotein.

**Fig 2:**
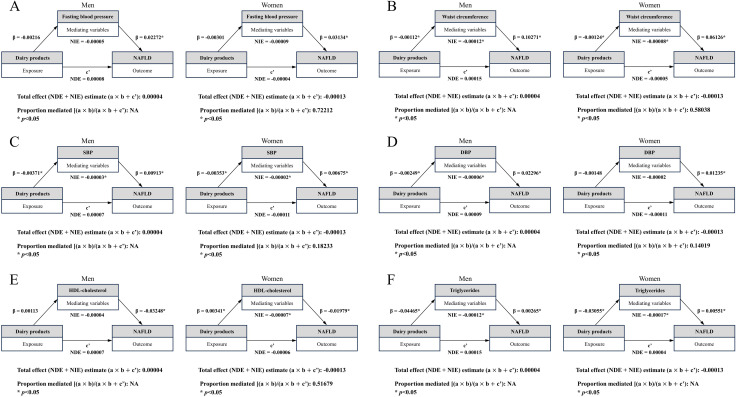
Model of the mediation relationship between dairy product consumption and nonalcoholic fatty liver disease through metabolic syndrome components as mediating variables. (A) fasting blood glucose. (B) Waist circumference. (C) SBP. (D) DBP. (E) HDL-cholesterol. (F) Triglycerides. The proportion mediated was calculated as the NIE divided by the total effect and was reported as NA when the value exceeded 1. All models were adjusted for age, BMI, education level, occupation, marital status, household income, household type, region, drinking, smoking, total energy intake, and physical activity. NAFLD, nonalcoholic fatty liver disease; NIE, natural indirect effect; NDE, natural direct effect; SBP, systolic blood pressure; DBP, diastolic blood pressure; HDL, high-density lipoprotein.

Fig 2 illustrates the mediating effects of MetS components on the association between dairy product intake and NAFLD. Among women, significant natural indirect effects (NIEs) of dairy product intake on NAFLD were observed through waist circumference (β = −0.00008; 95% CI −0.00012 to −0.00003), with a proportion mediated of 58.0% (Fig 2B), SBP (β = −0.00002; 95% CI −0.00004 to 0.0000), with a proportion mediated of 18.2% (Fig 2C), and through HDL cholesterol (β = −0.00007; 95% CI −0.00011 to −0.00003), with a proportion mediated of 51.7% (Fig 2E). The NIEs through fasting blood glucose (β = −0.00009; 95% CI −0.00020 to 0.00001) and DBP (β = −0.00002; 95% CI −0.00004 to 0.0000) were not statistically significant, although the corresponding proportions mediated were 72.2% and 14.0%, respectively (Figs 2A and 2D). No significant mediation was observed for triglyceride levels (Fig 2F). Among men, all mediated proportions were reported as not applicable (NA) because the estimated values exceeded 100%. Sensitivity analyses using age- and BMI-stratified mediation models were conducted, with a greater number of NIEs observed among women than men across strata (S1 and S2 Tables).

## Discussion

### Summary of study findings

This study examined the mediating effects of MetS components on the association between dairy product consumption and NAFLD in Korean adults. Among women, consumption of at least one serving of dairy products per day was associated with a lower prevalence of NAFLD as well as a reduced prevalence of several MetS components, including hyperglycemia, abdominal obesity, low HDL cholesterol, and elevated triglycerides, compared with women who did not consume dairy products. In contrast, no significant associations were observed among men. Additionally, all MetS components were independently associated with NAFLD in both men and women. Mediation analyses further indicated that the protective association between dairy intake and NAFLD in women was significantly mediated by waist circumference, SBP, and HDL cholesterol levels, whereas no significant mediation effects were observed among men.

### Association between dairy product consumption and NAFLD

The inverse association between dairy product consumption and NAFLD observed in this study is consistent with the findings of previous studies conducted in Asian populations. For example, the consumption of ≥ 50 g of full-fat dairy products was inversely associated with the prevalence of NAFLD in Thailand [18]. Similarly, among Chinese individuals, those who consumed seven servings of dairy products per week exhibited a lower incidence of NAFLD than those who did not consume dairy products [23].

Several biological mechanisms can explain this association. Dairy products with high calcium content can reduce the circulating levels of 1,25-dihydroxy vitamin D, resulting in limited uptake of calcium into cells. Lower intracellular calcium levels stimulate lipolysis and inhibit the expression of fatty acid synthase, which in turn inhibits lipid synthesis and prevents fat accumulation in the liver [13]. Additionally, dairy components such as inulin-type fructans and whey protein may modulate the gut microbiota, potentially contributing to the inhibition of NAFLD progression by regulating the gut-liver axis [24].

### Association between dairy product consumption and metabolic syndrome components

The relationship between dairy product consumption and MetS components has also been examined by previous studies [14,18,25,26]. A 200 g/day increase in milk consumption was associated with a 12% lower risk of abdominal obesity, and a 100 g/day increase in yogurt consumption was associated with a 16% lower risk of hyperglycemia [26]. In addition, when dairy product intake was divided into quartiles, individuals in the highest quartile had significantly lower odds of developing abdominal obesity and MetS than those in the lowest quartile [27].

This association can be attributed to several biological pathways. Whey proteins in dairy products increase plasma amino acid levels, including isoleucine, leucine, valine, and lysine [28]. This increase stimulates the production of glucagon-like peptide-1 and gastric inhibitory polypeptide [15], which subsequently enhances insulin secretion and leads to reduced blood glucose levels [16]. Furthermore, dietary calcium intake may increase HDL cholesterol by reducing lipid absorption via binding to bile acids and fatty acids [17,29]. Moreover, lactotripeptides in dairy proteins inhibit angiotensin-converting enzymes, thereby reducing blood pressure [30].

However, this study did not find a significant association between dairy product consumption and blood pressure. Previous studies have reported inconsistent results regarding this relationship. Nevertheless, several studies have identified an inverse association between dairy intake and blood pressure across diverse age groups, including adults, older adults, children, and adolescents [31–33]. Particularly, low-fat dairy consumption has been reported to be more effective in reducing blood pressure, whereas full-fat dairy products have shown little or no effect [34]. In contrast, a Dutch study reported that overall dairy consumption was not significantly associated with changes in blood pressure [35]. Moreover, a Mendelian randomization analysis incorporating data from 32 observational and cohort studies found no evidence of a causal effect of dairy consumption on SBP or the risk of hypertension [36]. Similarly, no significant association was observed between the consumption of different dairy products and the prevalence or incidence of hypertension [37]. Overall, this existing evidence does not provide a clear consensus on the relationship between dairy consumption and blood pressure. Therefore, further longitudinal and clinical studies are warranted to clarify the potential role of dairy consumption in the regulation of BP.

### Association between metabolic syndrome components and NAFLD

NAFLD and MetS are highly interrelated, with strong associations between NAFLD and individual components of MetS [38]. Insulin resistance disturbs blood glucose control, increases liver fat accumulation, and inhibits lipolysis, leading to increased very-low-density lipoprotein (VLDL) levels and decreased HDL cholesterol levels, thereby contributing to the progression of both MetS and NAFLD [39–41]. Elevated blood pressure is also associated with an increased risk of NAFLD, with individuals with prehypertension and hypertension showing approximately 1.3-fold and 1.8-fold higher odds of NAFLD, respectively, than those with normal blood pressure [42]. Moreover, abdominal obesity has been strongly associated with a higher risk of NAFLD [43], and individuals with NAFLD exhibit significantly higher triglyceride and lower HDL cholesterol levels than those without NAFLD [44].

### Sex-specific associations of dairy consumption with metabolic syndrome and NAFLD

The present study demonstrated that the associations between dairy consumption, NAFLD, and MetS components varied by sex, with significant associations observed only among women. Similar sex-specific patterns have been reported previously. A cohort study found that higher dairy consumption was strongly associated with a lower prevalence of NAFLD in women [45], a finding supported by a meta-analysis of observational studies that reported consistent results [46]. Regarding MetS components, a 10-year cohort study from the Korean Genome and Epidemiology Study (KoGES) showed that dairy consumption was associated with a reduced risk of low HDL-cholesterol among women [47], and a cross-sectional study using the KNHANES observed a lower MetS prevalence among Korean women consuming ≥1 serving/day of dairy products [25]. Moreover, MetS and its individual components are more strongly associated with NAFLD in women than in men [48].

These sex differences may partly arise because the metabolic effects of diets and dietary constituents are expressed in a sex-specific manner and highly dependent on the disease context [49]. Clinical evidence supports the existence of sex-specific metabolic responses to dairy consumption. In a 6-week randomized controlled trial (RCT), low-fat dairy intake differentially improved MetS parameters according to sex, with a modest but significant reduction in fasting blood glucose in men and reductions in waist circumference, body weight, and BMI in women [50]. Another RCT demonstrated distinct postprandial lipoprotein responses to dairy between sexes, with women exhibiting smaller increases in VLDL particles and greater increases in HDL particle concentrations than men [51]. Biological sex also strongly influences adipose tissue distribution and metabolic functions. Women generally have greater amounts of subcutaneous adipose tissue than men [52]. Although abdominal subcutaneous fat is associated with adverse glucose and lipid profiles [53], subcutaneous fat may provide partial protection against metabolic dysfunction, potentially reducing the risk of MetS in women [54]. In addition, insulin sensitivity differs by sex, with women typically exhibiting greater adipose tissue insulin sensitivity than men [55]. Dairy intake has consistently been shown to inhibit intestinal fat absorption, improve lipid profiles, reduce blood pressure, and enhance glucose metabolism [56–58], thereby contributing to improvements in NAFLD. Sex hormones further modulate these processes, with estrogens promoting metabolically protective pathways and androgens supporting male-specific metabolic environments [59]. Collectively, these biological and clinical differences suggest that dairy exposure may involve distinct metabolic pathways in men and women, contributing to the observed sex-specific associations.

### Sex-specific mediating effects of metabolic syndrome components in the dairy-NAFLD association

The present study found that the association between dairy consumption and NAFLD operates primarily through MetS components, rather than through a direct pathway. Additionally, in women, waist circumference, SBP, and HDL cholesterol appeared to serve as significant mediators of the dairy-NAFLD association, whereas no significant mediating effects were observed in men. This sex-specific mediation pattern supports the idea that the metabolic benefits of dairy intake are expressed differently according to sex. Women in this study consumed more dairy products than men, and consumption of more than one dairy product per day was significantly associated with lower odds of MetS in Korean women [25]. In addition, although dairy consumption did not differ between men in the NAFLD and non-NAFLD groups, women in the non-NAFLD group reported significantly higher dairy consumption than those with NAFLD. This pattern may explain why the beneficial metabolic effects of dairy intake, such as enhanced lipid excretion and lower blood glucose levels, are more pronounced in women. Importantly, the mediation analysis did not identify any significant direct effects of dairy consumption on NAFLD, indicating that dairy intake predominantly influences NAFLD through its effects on several metabolic dysfunctions, including elevated waist circumference, SBP, and low HDL cholesterol levels, rather than through a direct hepatic pathway in women.

### Study strengths and limitations

This study has certain limitations. First, it was a cross-sectional study using the KNHANES dataset, which does not allow for the establishment of a clear causal relationship. Additionally, although the KNHANES dataset includes information on whether participants were taking medication for dyslipidemia, it lacks detailed information regarding medication type, dosage, therapeutic targets (e.g., LDL cholesterol, HDL cholesterol, and triglycerides), and combination therapy. Moreover, the dataset does not provide information on liver fibrosis stage or liver enzyme levels, such as gamma-glutamyl transferase (GGT) or alkaline phosphatase (ALP), which may also influence NAFLD risk. Thus, additional longitudinal studies are needed to clarify the causal relationships among dairy products, NAFLD, and MetS. Furthermore, the use of noninvasive methods for diagnosing NAFLD may result in misclassification and diagnostic errors. Despite these limitations, this study has several strengths. To the best of our knowledge, this is the first study to identify the mediating effects of MetS components on the association between dairy product consumption and NAFLD. The serving sizes used in this study (200 g for milk, 100–150 g for yogurt, and 20 g for cheese) were consistent with the standard portions of international dietary guidelines [26,60,61] and represent realistic targets for clinical dietary counseling. Although dairy product preferences differ across countries, the focus on total dairy intake in the primary mediation analysis supports the broader applicability of our findings. Sensitivity analyses examining individual dairy types allow for interpretation across diverse dietary contexts, although the generalizability of cheese-specific findings may be limited, given the relatively low cheese consumption in our Korean cohort compared to Western populations.

## Conclusions

This study demonstrated that the associations between dairy product consumption and NAFLD and between dairy consumption and individual MetS components were significant among women but not among men. In contrast, the individual MetS components were significantly associated with NAFLD in both sexes. Mediation analyses further indicated that waist circumference, SBP, and HDL cholesterol levels mediated the association between dairy consumption and NAFLD in women, whereas no significant association was observed in men.

## Supporting information

S1 Table Mediation effects of metabolic syndrome components on the association between dairy product consumption and NAFLD, stratified by age. Statistically significant values are indicated in bold (*p < 0.05). β₁ represents the regression coefficient for the association between dairy product consumption and the metabolic syndrome component. β₂ represents the regression coefficient for the association between the metabolic syndrome component and NAFLD. The proportion mediated was calculated as NIE divided by the total effect and is reported as NA when the value exceeded 1. All models were adjusted for BMI, education level, occupation, marital status, household income, household type, region, drinking, smoking, total energy intake, and physical activity. NAFLD, non-alcoholic fatty liver disease; NIE, natural indirect effect; NDE, natural direct effect; SBP, systolic blood pressure; DBP, diastolic blood pressure; HDL, high-density lipoprotein.
(DOCX)

S2 Table Mediation effects of metabolic syndrome components on the association between dairy product consumption and NAFLD, stratified by BMI. Statistically significant values are indicated in bold (*p < 0.05). β₁ represents the regression coefficient for the association between dairy product consumption and the metabolic syndrome component. β₂ represents the regression coefficient for the association between the metabolic syndrome component and NAFLD. The proportion mediated was calculated as NIE divided by the total effect and is reported as NA when the value exceeded 1. All models were adjusted for age, education level, occupation, marital status, household income, household type, region, drinking, smoking, total energy intake, and physical activity. NAFLD, non-alcoholic fatty liver disease; NIE, natural indirect effect; NDE, natural direct effect; SBP, systolic blood pressure; DBP, diastolic blood pressure; HDL, high-density lipoprotein.
(DOCX)

## References

[1] Riazi K, Azhari H, Charette JH, Underwood FE, King JA, Afshar EE, et al. The prevalence and incidence of NAFLD worldwide: a systematic review and meta-analysis. Lancet Gastroenterol Hepatol. 2022;7(9):851–61. doi: 10.1016/S2468-1253(22)00165-0 35798021

[2] Im HJ, Ahn YC, Wang J-H, Lee MM, Son CG. Systematic review on the prevalence of nonalcoholic fatty liver disease in South Korea. Clin Res Hepatol Gastroenterol. 2021;45(4):101526. doi: 10.1016/j.clinre.2020.06.022 32919911

[3] Videla LA, Rodrigo R, Araya J, Poniachik J. Insulin resistance and oxidative stress interdependency in non-alcoholic fatty liver disease. Trends Mol Med. 2006;12(12):555–8. doi: 10.1016/j.molmed.2006.10.001 17049925

[4] Hamaguchi M, Kojima T, Takeda N, Nakagawa T, Taniguchi H, Fujii K, et al. The metabolic syndrome as a predictor of nonalcoholic fatty liver disease. Ann Intern Med. 2005;143(10):722–8. doi: 10.7326/0003-4819-143-10-200511150-00009 16287793

[5] Loomba R, Friedman SL, Shulman GI. Mechanisms and disease consequences of nonalcoholic fatty liver disease. Cell. 2021;184(10):2537–64. doi: 10.1016/j.cell.2021.04.015 33989548 PMC12168897

[6] Friedman SL, Neuschwander-Tetri BA, Rinella M, Sanyal AJ. Mechanisms of NAFLD development and therapeutic strategies. Nat Med. 2018;24(7):908–22. doi: 10.1038/s41591-018-0104-9 29967350 PMC6553468

[7] Fan J-G, Cao H-X. Role of diet and nutritional management in non-alcoholic fatty liver disease. J Gastroenterol Hepatol. 2013;28 Suppl 4:81–7. doi: 10.1111/jgh.12244 24251710

[8] Mouzaki M, Allard JP. The role of nutrients in the development, progression, and treatment of nonalcoholic fatty liver disease. J Clin Gastroenterol. 2012;46(6):457–67. doi: 10.1097/MCG.0b013e31824cf51e 22469640

[9] Wu H, Li S, Chen L, Xia Y, Tan X. Intake of the different types of dairy products, genetic predisposition, and the risks of nonalcoholic fatty liver disease and cirrhosis: a prospective cohort study. Food Funct. 2024;15(9):5050–62. doi: 10.1039/d3fo04602h 38656457

[10] He K, Li Y, Guo X, Zhong L, Tang S. Food groups and the likelihood of non-alcoholic fatty liver disease: a systematic review and meta-analysis. Br J Nutr. 2020;124(1):1–13. doi: 10.1017/S0007114520000914 32138796 PMC7512147

[11] Ebrahimi Mousavi S, Dehghanseresht N, Dashti F, Khazaei Y, Salamat S, Asbaghi O, et al. The association between Dietary Diversity Score and odds of nonalcoholic fatty liver disease: a case-control study. Eur J Gastroenterol Hepatol. 2022;34(6):678–85. doi: 10.1097/MEG.0000000000002344 35352692

[12] Petersen BL, Ward LS, Bastian ED, Jenkins AL, Campbell J, Vuksan V. A whey protein supplement decreases post-prandial glycemia. Nutr J. 2009;8:47. doi: 10.1186/1475-2891-8-47 19835582 PMC2766379

[13] van Meijl LEC, Vrolix R, Mensink RP. Dairy product consumption and the metabolic syndrome. Nutr Res Rev. 2008;21(2):148–57. doi: 10.1017/S0954422408116997 19087368

[14] Lee CJ, Joung H. Milk intake is associated with metabolic syndrome-using data from the Korea National Health and Nutrition Examination Survey 2007~ 2010. Korean J Community Nutr. 2012;17(6):795–804.

[15] Giezenaar C, Lange K, Hausken T, Jones KL, Horowitz M, Chapman I. Effects of age on acute appetite-related responses to whey-protein drinks, including energy intake, gastric emptying, blood glucose, and plasma gut hormone concentrations-a randomized controlled trial. Nutrients. 2020;12(4):1008. 32268554 10.3390/nu12041008 PMC7231005

[16] Gunnerud UJ, Ostman EM, Björck IME. Effects of whey proteins on glycaemia and insulinaemia to an oral glucose load in healthy adults; a dose-response study. Eur J Clin Nutr. 2013;67(7):749–53. doi: 10.1038/ejcn.2013.88 23632747

[17] Reid IR, Mason B, Horne A, Ames R, Clearwater J, Bava U, et al. Effects of calcium supplementation on serum lipid concentrations in normal older women: a randomized controlled trial. Am J Med. 2002;112(5):343–7. doi: 10.1016/s0002-9343(01)01138-x 11904107

[18] Charatcharoenwitthaya P, Tansakul E, Chaiyasoot K, Bandidniyamanon W, Charatcharoenwitthaya N. Dietary Composition and Its Association with Newly Diagnosed Nonalcoholic Fatty Liver Disease and Insulin Resistance. Nutrients. 2021;13(12):4438. doi: 10.3390/nu13124438 34959990 PMC8708546

[19] Hao X, Ma C, Xiang T, Ou L, Zeng Q. Associations Among Methylene Tetrahydrofolate Reductase rs1801133 C677T Gene Variant, Food Groups, and Non-alcoholic Fatty Liver Disease Risk in the Chinese Population. Front Genet. 2021;12:568398. doi: 10.3389/fgene.2021.568398 33679874 PMC7930608

[20] Ministry of Health and Welfare, The Korean Nutrition Society. Application of 2020 Dietary Reference Intakes for Koreans. 2021.

[21] Lee J-H, Kim D, Kim HJ, Lee C-H, Yang JI, Kim W, et al. Hepatic steatosis index: a simple screening tool reflecting nonalcoholic fatty liver disease. Dig Liver Dis. 2010;42(7):503–8. doi: 10.1016/j.dld.2009.08.002 19766548

[22] Expert Panel on Detection, Evaluation, and Treatment of High Blood Cholesterol in Adults. Executive Summary of The Third Report of The National Cholesterol Education Program (NCEP) Expert Panel on Detection, Evaluation, And Treatment of High Blood Cholesterol In Adults (Adult Treatment Panel III). JAMA. 2001;285(19):2486–97. doi: 10.1001/jama.285.19.2486 11368702

[23] Xu Y, Wang Y, Zhao Q, Chen B, Wang N, Zhang T, et al. Dairy products intake and prevalence, incidence, and recovery of non-alcoholic fatty liver disease in Chinese population. Hepatol Int. 2024;18(2):529–39. doi: 10.1007/s12072-024-10638-w 38409495

[24] Reimer RA, Willis HJ, Tunnicliffe JM, Park H, Madsen KL, Soto-Vaca A. Inulin-type fructans and whey protein both modulate appetite but only fructans alter gut microbiota in adults with overweight/obesity: A randomized controlled trial. Mol Nutr Food Res. 2017;61(11):10.1002/mnfr.201700484. doi: 10.1002/mnfr.201700484 28730743

[25] Lee KW, Cho W. The Consumption of Dairy Products Is Associated with Reduced Risks of Obesity and Metabolic Syndrome in Korean Women but not in Men. Nutrients. 2017;9(6):630. doi: 10.3390/nu9060630 28629203 PMC5490609

[26] Lee M, Lee H, Kim J. Dairy food consumption is associated with a lower risk of the metabolic syndrome and its components: a systematic review and meta-analysis. Br J Nutr. 2018;120(4):373–84. doi: 10.1017/S0007114518001460 29871703

[27] Azadbakht L, Mirmiran P, Esmaillzadeh A, Azizi F. Dairy consumption is inversely associated with the prevalence of the metabolic syndrome in Tehranian adults. Am J Clin Nutr. 2005;82(3):523–30. doi: 10.1093/ajcn.82.3.523 16155263

[28] Nilsson M, Stenberg M, Frid AH, Holst JJ, Björck IME. Glycemia and insulinemia in healthy subjects after lactose-equivalent meals of milk and other food proteins: the role of plasma amino acids and incretins. Am J Clin Nutr. 2004;80(5):1246–53. doi: 10.1093/ajcn/80.5.1246 15531672

[29] Ohlsson L. Dairy products and plasma cholesterol levels. Food Nutr Res. 2010;54(1):5124. doi: 10.3402/fnr.v54i0.5124 20806084 PMC2926059

[30] McGrane MM, Essery E, Obbagy J, Lyon J, Macneil P, Spahn J, et al. Dairy Consumption, Blood Pressure, and Risk of Hypertension: An Evidence-Based Review of Recent Literature. Curr Cardiovasc Risk Rep. 2011;5(4):287–98. doi: 10.1007/s12170-011-0181-5 22384284 PMC3289141

[31] Beydoun MA, Gary TL, Caballero BH, Lawrence RS, Cheskin LJ, Wang Y. Ethnic differences in dairy and related nutrient consumption among US adults and their association with obesity, central obesity, and the metabolic syndrome. Am J Clin Nutr. 2008;87(6):1914–25. doi: 10.1093/ajcn/87.6.1914 18541585 PMC2585752

[32] Lana A, Banegas JR, Guallar-Castillón P, Rodríguez-Artalejo F, Lopez-Garcia E. Association of Dairy Consumption and 24-Hour Blood Pressure in Older Adults with Hypertension. Am J Med. 2018;131(10):1238–49. doi: 10.1016/j.amjmed.2018.04.039 29807002

[33] Yuan WL, Kakinami L, Gray-Donald K, Czernichow S, Lambert M, Paradis G. Influence of dairy product consumption on children’s blood pressure: results from the QUALITY cohort. J Acad Nutr Diet. 2013;113(7):936–41. doi: 10.1016/j.jand.2013.03.010 23684662

[34] Alonso A, Beunza JJ, Delgado-Rodríguez M, Martínez JA, Martínez-González MA. Low-fat dairy consumption and reduced risk of hypertension: the Seguimiento Universidad de Navarra (SUN) cohort. Am J Clin Nutr. 2005;82(5):972–9. doi: 10.1093/ajcn/82.5.972 16280427

[35] Engberink MF, Geleijnse JM, de Jong N, Smit HA, Kok FJ, Verschuren WMM. Dairy intake, blood pressure, and incident hypertension in a general Dutch population. J Nutr. 2009;139(3):582–7. doi: 10.3945/jn.108.093088 19158223

[36] Ding M, Huang T, Bergholdt HK, Nordestgaard BG, Ellervik C, Qi L, et al. Dairy consumption, systolic blood pressure, and risk of hypertension: Mendelian randomization study. BMJ. 2017;356:j1000. doi: 10.1136/bmj.j1000 28302601 PMC6168037

[37] Farinha VO, Vaucher J, Vidal P-M. Dairy products and hypertension: Cross-sectional and prospective associations. Clin Nutr ESPEN. 2024;63:597–603. doi: 10.1016/j.clnesp.2024.07.020 39067698

[38] Andappa JM. Prevalence of metabolic syndrome and its association with non-alcoholic fatty liver disease in adults: A cross-sectional study. EJCM. 2023;13:2394–9.

[39] Goetze S, Kim S, Xi XP, Graf K, Yang DC, Fleck E, et al. Troglitazone inhibits mitogenic signaling by insulin in vascular smooth muscle cells. J Cardiovasc Pharmacol. 2000;35(5):749–57. doi: 10.1097/00005344-200005000-00011 10813377

[40] Bugianesi E, Moscatiello S, Ciaravella MF, Marchesini G. Insulin resistance in nonalcoholic fatty liver disease. Curr Pharm Des. 2010;16(17):1941–51. doi: 10.2174/138161210791208875 20370677

[41] Adiels M, Olofsson S-O, Taskinen M-R, Borén J. Overproduction of very low-density lipoproteins is the hallmark of the dyslipidemia in the metabolic syndrome. Arterioscler Thromb Vasc Biol. 2008;28(7):1225–36. doi: 10.1161/ATVBAHA.107.160192 18565848

[42] Aneni EC, Oni ET, Martin SS, Blaha MJ, Agatston AS, Feldman T, et al. Blood pressure is associated with the presence and severity of nonalcoholic fatty liver disease across the spectrum of cardiometabolic risk. J Hypertens. 2015;33(6):1207–14. doi: 10.1097/HJH.0000000000000532 25693058

[43] Kuang M, Lu S, Xie Q, Peng N, He S, Yu C, et al. Abdominal obesity phenotypes are associated with the risk of developing non-alcoholic fatty liver disease: insights from the general population. BMC Gastroenterol. 2022;22(1):311. doi: 10.1186/s12876-022-02393-9 35752753 PMC9233393

[44] Hoca E, Cangir B, Ahbab S, Şahin Sİ, Çiftçi Öztürk E, Urvasızoğlu AÖ, et al. The Triglyceride/HDL Ratio as a Non-Invasive Marker for Early-Stage NAFLD: A Retrospective Cross-Sectional Study of 2588 Patients. Diagnostics (Basel). 2025;15(16):2045. doi: 10.3390/diagnostics15162045 40870897 PMC12385797

[45] Keshavarz Z, Rahimlou M, Farjam M, Homayounfar R, Khodadost M, Abdollahi A, et al. Non-alcoholic fatty liver disease and dairy products consumption: Results from FASA Persian cohort study. Front Nutr. 2022;9:962834. doi: 10.3389/fnut.2022.962834 36159487 PMC9500577

[46] Dai W, Liu H, Zhang T, Chang Q, Zhao Y, Guo C, et al. Dairy product consumption was associated with a lower likelihood of non-alcoholic fatty liver disease: A systematic review and meta-analysis. Front Nutr. 2023;10:1119118. doi: 10.3389/fnut.2023.1119118 36908921 PMC9992538

[47] Kim D, Kim J. Dairy consumption is associated with a lower incidence of the metabolic syndrome in middle-aged and older Korean adults: the Korean Genome and Epidemiology Study (KoGES). Br J Nutr. 2017;117(1):148–60. doi: 10.1017/S000711451600444X 28098053

[48] Lin I-T, Lee M-Y, Wang C-W, Wu D-W, Chen S-C. Gender Differences in the Relationships among Metabolic Syndrome and Various Obesity-Related Indices with Nonalcoholic Fatty Liver Disease in a Taiwanese Population. Int J Environ Res Public Health. 2021;18(3):857. doi: 10.3390/ijerph18030857 33498329 PMC7908550

[49] Pontifex MG, Vauzour D, Muller M. Sexual dimorphism in the context of nutrition and health. Proc Nutr Soc. 2024;83(2):109–19. doi: 10.1017/S0029665123003610 37665115

[50] Dugan CE, Barona J, Fernandez ML. Increased dairy consumption differentially improves metabolic syndrome markers in male and female adults. Metab Syndr Relat Disord. 2014;12(1):62–9. doi: 10.1089/met.2013.0109 24236646

[51] Hansson P, Holven KB, Øyri LKL, Brekke HK, Gjevestad GO, Thoresen M, et al. Sex differences in postprandial responses to different dairy products on lipoprotein subclasses: a randomised controlled cross-over trial. Br J Nutr. 2019;122(7):780–9. doi: 10.1017/S0007114519001429 31208475

[52] Bredella MA. Sex Differences in Body Composition. Adv Exp Med Biol. 2017;1043:9–27. doi: 10.1007/978-3-319-70178-3_2 29224088

[53] Snijder MB, Visser M, Dekker JM, Goodpaster BH, Harris TB, Kritchevsky SB, et al. Low subcutaneous thigh fat is a risk factor for unfavourable glucose and lipid levels, independently of high abdominal fat. The Health ABC Study. Diabetologia. 2005;48(2):301–8. doi: 10.1007/s00125-004-1637-7 15660262

[54] Bergman RN, Kim SP, Catalano KJ, Hsu IR, Chiu JD, Kabir M, et al. Why visceral fat is bad: mechanisms of the metabolic syndrome. Obesity (Silver Spring). 2006;14(2S):16S–19S. doi: 10.1038/oby.2006.277 16642958

[55] Geer EB, Shen W. Gender differences in insulin resistance, body composition, and energy balance. Gend Med. 2009;6(Suppl 1):60–75. doi: 10.1016/j.genm.2009.02.002 19318219 PMC2908522

[56] Govers MJ, Termont DS, Lapré JA, Kleibeuker JH, Vonk RJ, Van der Meer R. Calcium in milk products precipitates intestinal fatty acids and secondary bile acids and thus inhibits colonic cytotoxicity in humans. Cancer Res. 1996;56(14):3270–5. 8764120

[57] Foltz M, Meynen EE, Bianco V, van Platerink C, Koning TMMG, Kloek J. Angiotensin converting enzyme inhibitory peptides from a lactotripeptide-enriched milk beverage are absorbed intact into the circulation. J Nutr. 2007;137(4):953–8. doi: 10.1093/jn/137.4.953 17374660

[58] O’Connor S, Turcotte A-F, Gagnon C, Rudkowska I. Increased Dairy Product Intake Modifies Plasma Glucose Concentrations and Glycated Hemoglobin: A Systematic Review and Meta-Analysis of Randomized Controlled Trials. Adv Nutr. 2019;10(2):262–79. doi: 10.1093/advances/nmy074 30657868 PMC6416042

[59] Kim YS, Unno T, Kim BY, Park MS. Sex Differences in Gut Microbiota. World J Mens Health. 2020;38(1):48–60. doi: 10.5534/wjmh.190009 30929328 PMC6920072

[60] Chen Z, Ahmed M, Ha V, Jefferson K, Malik V, Ribeiro PAB, et al. Dairy Product Consumption and Cardiovascular Health: A Systematic Review and Meta-analysis of Prospective Cohort Studies. Adv Nutr. 2022;13(2):439–54. doi: 10.1093/advances/nmab118 34550320 PMC8970833

[61] European Commission Health Promotion Knowledge Gateway. Food-Based Dietary Guidelines recommendations for milk and dairy products for the EU, Iceland, Norway, Switzerland and the United Kingdom. Knowledge4Policy. Updated Nov 7 2025. Accessed [Dec 31 2025].

