# Supplementary material for: Examining the mediating effects of metabolic syndrome components on the relationship between dairy product consumption and nonalcoholic fatty liver disease in Korean adults
Source: PLoS One. 2026 Apr 10;21(4):e0346774. doi: 10.1371/journal.pone.0346774 (PMC13068242; doi:10.1371/journal.pone.0346774)
Supplement: S2 Table — Statistically significant values are indicated in bold (*p < 0.05). β₁ represents the regression coefficient for the association between dairy product consumption and the metabolic syndrome component. β₂ represents the regression coefficient for the association between the metabolic syndrome component and NAFLD. The proportion mediated was calculated as NIE divided by the total effect and is reported as NA when the value exceeded 1. All models were adjusted for age, education level, occupation, marital status, household income, household type, region, drinking, smoking, total energy intake, and physical activity. NAFLD, non-alcoholic fatty liver disease; NIE, natural indirect effect; NDE, natural direct effect; SBP, systolic blood pressure; DBP, diastolic blood pressure; HDL, high-density lipoprotein. (DOCX) [file pone.0346774.s002.docx]

**S2 table. Mediation effects of metabolic syndrome components on the association between dairy product consumption and NAFLD, stratified by BMI**

|  | BMI (kg/m^2^) | Dairy → mediator (β₁) | Mediator → NAFLD (β₂) | NIE | NDE | Total effect | Proportion mediated |
| --- | --- | --- | --- | --- | --- | --- | --- |
| *Men* |  |  |  |  |  |  |  |
| Fasting blood glucose | <18.5 | -0.00534 | 0.02086 | -0.00011 | -0.00050 | -0.00061 | 0.18230 |
|  | 18.5-22.9 | -0.00525 | **0.03651*** | -0.00019 | 0.00041 | 0.00022 | -0.87601 |
|  | 23.0-24.9 | 0.00132 | **0.02393*** | 0.00003 | -0.00017 | -0.00014 | -0.22524 |
|  | ≥25 | -0.00249 | **0.03617*** | -0.00009 | -0.00077 | -0.00086 | 0.10501 |
| Waist circumference | <18.5 | -0.00208 | **0.12025*** | -0.00025 | -0.00036 | -0.00061 | 0.40907 |
|  | 18.5-22.9 | -0.00144 | **0.26849*** | -0.00039 | 0.00060 | 0.00022 | NA |
|  | 23.0-24.9 | -0.00135 | **0.18938*** | -0.00026 | 0.00012 | -0.00014 | NA |
|  | ≥25 | **-0.00264*** | **0.39727*** | **-0.00105*** | 0.00019 | -0.00086 | NA |
| SBP | <18.5 | -0.00294 | 0.02099 | -0.00006 | -0.00055 | -0.00061 | 0.10116 |
|  | 18.5-22.9 | -0.00373 | 0.00904 | -0.00003 | 0.00025 | 0.00022 | -0.15414 |
|  | 23.0-24.9 | -0.00489 | 0.00910 | -0.00004 | -0.00010 | -0.00014 | 0.31851 |
|  | ≥25 | **-0.00393*** | **0.04209*** | -0.00017 | -0.00069 | -0.00086 | 0.19288 |
| DBP | <18.5 | -0.00243 | 0.03368 | -0.00008 | -0.00053 | -0.00061 | 0.13389 |
|  | 18.5-22.9 | -0.00171 | **0.01659*** | -0.00003 | 0.00025 | 0.00022 | -0.12962 |
|  | 23.0-24.9 | **-0.00418*** | **0.02939*** | -0.00012 | -0.00002 | -0.00014 | 0.87830 |
|  | ≥25 | -0.00225 | **0.06730*** | -0.00015 | -0.00071 | -0.00086 | 0.17686 |
| HDL-cholesterol | <18.5 | -0.00435 | -0.00171 | 0.00001 | -0.00062 | -0.00061 | -0.01221 |
|  | 18.5-22.9 | 0.00118 | **-0.04893*** | -0.00006 | 0.00028 | 0.00022 | -0.26350 |
|  | 23.0-24.9 | 0.00246 | **-0.02254*** | -0.00006 | -0.00008 | -0.00014 | 0.39699 |
|  | ≥25 | 0.00112 | **-0.08353*** | -0.00009 | -0.00076 | -0.00086 | 0.10867 |
| Triglycerides | <18.5 | -0.01174 | 0.00136 | -0.00002 | -0.00059 | -0.00061 | 0.02612 |
|  | 18.5-22.9 | -0.03411 | **0.00354*** | -0.00012 | 0.00034 | 0.00022 | -0.55238 |
|  | 23.0-24.9 | -0.02678 | **0.00257*** | -0.00007 | -0.00007 | -0.00014 | 0.49329 |
|  | ≥25 | **-0.06365*** | **0.00417*** | **-0.00027*** | -0.00059 | -0.00086 | 0.30988 |
| *Women* |  |  |  |  |  |  |  |
| Fasting blood glucose | <18.5 | -0.00009 | **0.03867*** | 0.00000 | 0.00144 | 0.00144 | -0.00231 |
|  | 18.5-22.9 | -0.00352 | **0.03619*** | -0.00013 | -0.00007 | -0.00020 | 0.63085 |
|  | 23.0-24.9 | -0.00699 | **0.03674*** | -0.00026 | 0.00010 | -0.00015 | NA |
|  | ≥25 | 0.00048 | **0.04421*** | 0.00002 | -0.00067 | -0.00065 | -0.03253 |
| Waist circumference | <18.5 | 0.00002 | **0.11913*** | 0.00000 | 0.00144 | 0.00144 | 0.00198 |
|  | 18.5-22.9 | **-0.00141*** | **0.22519*** | **-0.00032*** | 0.00012 | -0.00020 | NA |
|  | 23.0-24.9 | **-0.00187*** | **0.10469*** | **-0.00020*** | 0.00004 | -0.00015 | NA |
|  | ≥25 | -0.00163 | **0.40088*** | -0.00066 | 0.00001 | -0.00065 | NA |
| SBP | <18.5 | 0.00008 | -0.01069 | 0.00000 | 0.00144 | 0.00144 | -0.00062 |
|  | 18.5-22.9 | **-0.00445*** | **0.00781*** | -0.00003 | -0.00017 | -0.00020 | 0.17191 |
|  | 23.0-24.9 | -0.00004 | **0.01190*** | 0.00000 | -0.00015 | -0.00015 | 0.00317 |
|  | ≥25 | -0.00471 | **0.04477*** | -0.00021 | -0.00044 | -0.00065 | 0.32650 |
| DBP | <18.5 | 0.00040 | -0.01643 | -0.00001 | 0.00145 | 0.00144 | -0.00460 |
|  | 18.5-22.9 | **-0.00236*** | 0.00833 | -0.00002 | -0.00018 | -0.00020 | 0.09727 |
|  | 23.0-24.9 | 0.00077 | **0.02216*** | 0.00002 | -0.00017 | -0.00015 | -0.11191 |
|  | ≥25 | -0.00122 | **0.07555*** | -0.00009 | -0.00055 | -0.00065 | 0.14266 |
| HDL-cholesterol | <18.5 | 0.00953 | -0.01071 | -0.00010 | 0.00154 | 0.00144 | -0.07086 |
|  | 18.5-22.9 | **0.00353*** | **-0.02847*** | **-0.00010*** | -0.00010 | -0.00020 | 0.49741 |
|  | 23.0-24.9 | -0.00035 | **-0.02511*** | 0.00001 | -0.00016 | -0.00015 | -0.05761 |
|  | ≥25 | **0.00511*** | **-0.05482*** | **-0.00028*** | -0.00037 | -0.00065 | 0.43360 |
| Triglycerides | <18.5 | **-0.03776*** | 0.00536 | -0.00020 | **0.00164*** | 0.00144 | -0.14069 |
|  | 18.5-22.9 | **-0.01567*** | **0.00715*** | **-0.00011*** | -0.00009 | -0.00020 | 0.55494 |
|  | 23.0-24.9 | -0.02664 | **0.00633*** | -0.00017 | 0.00002 | -0.00015 | NA |
|  | ≥25 | **-0.05846*** | **0.00951*** | **-0.00056*** | -0.00009 | -0.00065 | 0.86062 |

Statistically significant values are indicated in bold **(**p* < 0.05**).

β₁ represents the regression coefficient for the association between dairy product consumption and the metabolic syndrome component.

β₂ represents the regression coefficient for the association between the metabolic syndrome component and NAFLD.

The proportion mediated was calculated as NIE divided by the total effect and is reported as NA when the value exceeded 1.

All models were adjusted for age, education level, occupation, marital status, household income, household type, region, drinking, smoking, total energy intake, and physical activity.

NAFLD, non-alcoholic fatty liver disease; NIE, natural indirect effect; NDE, natural direct effect; SBP, systolic blood pressure; DBP, diastolic blood pressure; HDL, high-density lipoprotein.
