# Supplementary material for: Examining the mediating effects of metabolic syndrome components on the relationship between dairy product consumption and nonalcoholic fatty liver disease in Korean adults
Source: PLoS One. 2026 Apr 10;21(4):e0346774. doi: 10.1371/journal.pone.0346774 (PMC13068242; doi:10.1371/journal.pone.0346774)
Supplement: Attachment — Submitted filename: Response to Reviewers_0320.docx [file pone.0346774.s004.docx]

**Response letter to Reviewer 1**

1) What is Your mean of Mediation analysis in key words?
***Response:*** The authors thank the reviewer’s comment.

Mediation analysis examines the underlying mechanism of the observed relationship between an exposure variable and an outcome variable, exploring how these two variables are related through a third intermediate variable called the mediator. Rather than assuming a direct causal relationship between the independent and dependent variables, mediation analysis hypothesizes that the exposure variable influences the mediator, which in turn affects the outcome variable. Through this process, the mediator clarifies the nature of the relationship between the exposure and outcome variables.

Reference: MacKinnon, D. (2012). *Introduction to statistical mediation analysis*. Routledge.

2) Please mention mechanisms of dairy effects on metabolic syndrome and NAFLD in the abstract. Also add a brief review of previous study.

***Response:*** Thank you very much for your suggestion. Abstract was altered to the following.

It is now read as, “Calcium in dairy products improves the blood lipid profile by increasing HDL cholesterol and reducing triglycerides, while also promoting fat excretion to reduce liver fat accumulation. Additionally, whey protein enhances insulin secretion and improves lipid metabolism in the liver, thereby reducing fat accumulation. Whey protein also reduces postprandial blood glucose levels and suppresses appetite, mitigating key risk factors of metabolic syndrome (MetS). Additionally, probiotics in dairy products improve insulin resistance, while calcium increases HDL cholesterol and helps lower blood pressure. The mechanisms of dairy product consumption affect the MetS components and thereby influence non-alcoholic fatty liver disease (NAFLD) remain unclear. This study aimed to examine the mediating effects of MetS components on dairy product consumption and NAFLD.”

Reference: Soerensen, K.V., et al., Effect of dairy calcium from cheese and milk on fecal fat excretion, blood lipids, and appetite in young men. AJCN, 2014. 99(5): p. 984-991.

Tulipano, G., et al., Whey proteins as source of dipeptidyl dipeptidase IV (dipeptidyl peptidase-4) inhibitors. Peptides, 2011. 32(4): p. 835-838.

Azadbakht, L., et al., Dairy consumption is inversely associated with the prevalence of the metabolic syndrome in Tehranian adults. Am J Clin Nutr, 2005. 82(3): p. 523-30.

Petersen, B.L., et al., A whey protein supplement decreases post-prandial glycemia. Nutr. J., 2009. 8: p. 1-5.

Akhavan, T., et al., Effect of premeal consumption of whey protein and its hydrolysate on food intake and postmeal glycemia and insulin responses in young adults. AJCN, 2010. 91(4): p. 966-975.

van Meijl, L.E., R. Vrolix, and R.P. Mensink, Dairy product consumption and the metabolic syndrome. Nutr. Res. Rev., 2008. 21(2): p. 148-157.

Hajjar, I.M., C.E. Grim, and T.A. Kotchen, Dietary calcium lowers the age‐related rise in blood pressure in the United States: the NHANES III survey. J. Clin. Hypertens., 2003. 5(2): p. 122-126.

Jorde, R. and K.H. Bønaa, Calcium from dairy products, vitamin D intake, and blood pressure: the Tromsø study. Am. J. Clin., 2000. 71(6): p. 1530-1535.

3) It was better you using the 24-h dietary recall third a week (One holiday and two non-holiday days.

***Response:*** The authors greatly appreciate the reviewer’s recommendation.

As mentioned in the Methods and Discussion limitations, the study used the KNHANES dataset, so the authors were not able to change the day of the survey at their discretion. In KNHANES, participants completed the 24-hour dietary recall for only one day.

4) Please add statistical methods under all tables.

***Response:*** The authors greatly appreciate the response. We added statistical methods under all tables (Tables 3-5).

| **Table 3. Association between dairy products intake and non-alcoholic fatty liver disease (NAFLD)** | | | | | |
| --- | --- | --- | --- | --- | --- |
| **Men (n=5,492)** | **AOR (95% CI)^1^** | ***p*-value** | **Women (n=7,283)** | **AOR (95% CI)** | ***p*-value** |
| **Dairy products (serving size)** | | | **Dairy products (serving size)** | | |
| 0 (n=3,506) | 1.00 (Ref.) | | 0 (n=3,953) | 1.00 (Ref.) | |
| 0 to 1 (n=656) | 1.07 (0.79–1.46) | 0.66 | 0 to 1 (n=1,113) | 0.88 (0.66–1.17) | 0.39 |
| ≥ 1 (n=1,330) | 1.02 (0.81–1.28) | 0.89 | ≥ 1 (n=2,217) | 0.75 (0.59–0.96) | 0.02 |
| **Men (n = 5,492)** | **β ± SE** | **p-value** | **Women (n = 7,238)** | **β ± SE** | **p-value** |
| Dairy products (g/day) | -0.0001 ± 0.0003 | 0.4349 | Dairy products (g/day) | -0.0002 ± 0.0002 | 0.3898 |
| Types of dairy products |  |  | Types of dairy products |  |  |
| Milk (g/day) | -0.0001 ± 0.0003 | 0.8042 | Milk (g/day) | -0.0002 ± 0.0002 | 0.3727 |
| Liquid yogurt (g/day) | -0.0017 ± 0.0016 | 0.2821 | Liquid yogurt (g/day) | 0.0002 ± 0.0012 | 0.8159 |
| Semisolid yogurt (g/day) | -0.0002 ± 0.0008 | 0.8085 | Semisolid yogurt (g/day) | -0.00002 ± 0.0005 | 0.9676 |
| Ice cream (g/day) | 0.0005 ± 0.0012 | 0.7054 | Ice cream (g/day) | -0.0005 ± 0.0014 | 0.7009 |
| Cheese (g/day) | 0.0003 ± 0.0072 | 0.9619 | Cheese (g/day) | -0.0005 ± 0.0030 | 0.8609 |
| Cream (g/day) | -0.0004 ± 0.007 | 0.9517 | Cream (g/day) | 0.001 ± 0.003 | 0.7928 |
| One serving size of dairy products was defined as 200g for milk, 150g for liquid yogurt, 100 g for semisolid yogurt, ice cream, and cream and 20 g for cheese. Dairy product intake was classified “0” for never intake, “0–1” for exceed 0g and intake of less than one serving size, and “≥1” for intake of more than one serving size. | | | | | |
| ^1^Adjusted for age, BMI, education level, occupation, marital status, household income, household type, region, drinking, smoking, total energy intake, and physical activity. | | | | | |
| Multivariable logistic regression and linear regression were used to estimate the association between dairy products intake and NAFLD. | | | | | |
| Abbreviations: NAFLD, non-alcoholic fatty liver disease; AOR, adjusted odds ratio; CI, confidence interval; SE, standard error. | | | | | |

| **Table 4. Association between dairy products intake and components of metabolic syndrome** | | | | | | | | | | | | | | | | | | | | | |
| --- | --- | --- | --- | --- | --- | --- | --- | --- | --- | --- | --- | --- | --- | --- | --- | --- | --- | --- | --- | --- | --- |
|  | | **Hyperglycemia** | | | | **Abdominal obesity** | | | | **Elevated blood pressure** | | | | **Low HDL-cholesterol** | | | | **Elevated triglycerides** | | | |
| **Men (n = 5,492)** | | **AOR**^1^ **(95% CI)** | | ***p*-value** | | **AOR (95% CI)** | | ***p*-value** | | **AOR (95% CI)** | | | ***p*-value** | **AOR (95% CI)** | | ***p*-value** | | **AOR (95% CI)** | | ***p*-value** | |
| **Dairy products (serving size)** | | | |  | |  | |  | |  | | |  |  | |  | |  | |  | |
| 0 (n=3,520) | | 1.00 (Ref.) | | | | 1.00 (Ref.) | | | | 1.00 (Ref.) | | | | 1.00 (Ref.) | | | | 1.00 (Ref.) | | | |
| 0 to 1 (n=658) | | 1.04 (0.83–1.30) | | 0.72 | | 1.03 (0.82–1.28) | | 0.83 | | 0.95 (0.76–1.19) | | | 0.65 | 1.08 (0.84–1.39) | | 0.53 | | 0.98 (0.79–1.20) | | 0.81 | |
| ≥ 1 (n=1,340) | | 0.88 (0.74–1.03) | | 0.11 | | 0.87 (0.74–1.03) | | 0.10 | | 0.83 (0.69–1.01) | | | 0.06 | 0.93 (0.78–1.11) | | 0.40 | | 0.75 (0.63–0.89) | | 0.001 | |
| **Women (n=7,238)** | | **AOR (95% CI)** | | ***p*-value** | | **AOR (95% CI)** | | ***p*-value** | | **AOR (95% CI)** | | | ***p*-value** | **AOR (95% CI)** | | ***p*-value** | | **AOR (95% CI)** | | ***p*-value** | |
| **Dairy products (serving size)** | | | |  | |  | |  | |  | | |  |  | |  | |  | |  | |
| 0 (n=3,982) | | 1.00 (Ref.) | | | | 1.00 (Ref.) | | | | 1.00 (Ref.) | | | | 1.00 (Ref.) | | | | 1.00 (Ref.) | | | |
| 0 to 1 (n=1,120) | | 0.83 (0.69–1.01) | | 0.06 | | 0.83 (0.69–1.01) | | 0.07 | | 0.92 (0.75–1.14) | | | 0.43 | 0.79 (0.65–0.96) | | 0.02 | | 0.84 (0.69–1.01) | | 0.07 | |
| ≥ 1 (n=2,236) | | 0.84 (0.73–0.96) | | 0.02 | | 0.84 (0.73–0.97) | | 0.02 | | 0.96 (0.81–1.13) | | | 0.60 | 0.83 (0.72–0.95) | | 0.01 | | 0.71 (0.60–0.85) | | 0.0001 | |
|  | **Fasting blood glucose (mg/dL)** | | | | **Waist circumference (cm)** | | | | **SBP (mmHg)** | | | **DBP (mmHg)** | | | **HDL cholesterol (mg/dL)** | | | | **Triglycerides (mg/dL)** | | |
| **Men (n = 5,492)** | **β ± SE** | | ***p*-value** | | **β ± SE** | | ***p*-value** | | **β ± SE** | | ***p*-value** | **β ± SE** | | ***p*-value** | **β ± SE** | | ***p*-value** | | **β ± SE** | | ***p*-value** |
| Dairy products (g/day) | -0.002 ± 0.002 | | 0.49 | | -0.0009 ± 0.0004 | | 0.02 | | -0.003 ± 0.001 | | 0.02 | -0.002 ± 0.001 | | 0.02 | 0.0005 ± 0.001 | | 0.68 | | -0.037 ± 0.010 | | 0.0003 |
| Milk (g/day) | -0.002 ± 0.003 | | 0.42 | | -0.001 ± 0.0005 | | 0.22 | | -0.002 ± 0.001 | | 0.22 | -0.003 ± 0.001 | | 0.02 | 0.001 ± 0.001 | | 0.59 | | -0.040 ± 0.010 | | 0.0001 |
| Liquid yogurt (g/day) | -0.025 ± 0.009 | | 0.004 | | -0.006 ± 0.002 | | 0.01 | | -0.020 ± 0.008 | | 0.01 | 0.002 ± 0.009 | | 0.83 | -0.001 ± 0.006 | | 0.83 | | 0.092 ± 0.100 | | 0.35 |
| Semisolid yogurt (g/day) | 0.0005 ± 0.006 | | 0.93 | | -0.002 ± 0.001 | | 0.05 | | -0.003 ± 0.004 | | 0.42 | 0.001 ± 0.003 | | 0.76 | 0.001 ± 0.003 | | 0.73 | | -0.057 ± 0.032 | | 0.07 |
| Ice cream (g/day) | 0.016 ± 0.014 | | 0.23 | | -0.001 ± 0.002 | | 0.54 | | -0.017 ± 0.005 | | 0.002 | -0.007 ± 0.004 | | 0.11 | -0.004 ± 0.005 | | 0.34 | | -0.008 ± 0.046 | | 0.86 |
| Cheese(g/day) | -0.035 ± 0.018 | | 0.05 | | -0.006 ± 0.005 | | 0.27 | | 0.011 ± 0.014 | | 0.46 | -0.012 ± 0.015 | | 0.43 | -0.001 ± 0.014 | | 0.93 | | -0.253 ± 0.135 | | 0.06 |
| Cream (g/day) | -0.052 ± 0.014 | | 0.0001 | | -0.002 ± 0.006 | | 0.72 | | 0.024 ± 0.020 | | 0.23 | 0.019 ± 0.014 | | 0.16 | 0.034 ± 0.020 | | 0.10 | | -0.110 ± 0.186 | | 0.56 |
|  | **Fasting blood glucose (mg/dL)** | | | | **Waist circumference (cm)** | | | | **SBP (mmHg)** | | | **DBP (mmHg)** | | | **HDL cholesterol (mg/dL)** | | | | **Triglycerides (mg/dL)** | | |
| **Women (n = 7238)** | **β ± SE** | | ***p*-value** | | **β ± SE** | | ***p*-value** | | **β ± SE** | | ***p*-value** | **β ± SE** | | ***p*-value** | **β ± SE** | | ***p*-value** | | **β ± SE** | | ***p*-value** |
| Dairy products (g/day) | -0.002 ± 0.002 | | 0.16 | | -0.001 ± 0.0004 | | 0.03 | | -0.004 ± 0.001 | | 0.01 | -0.001 ± 0.001 | | 0.23 | 0.003 ± 0.001 | | 0.01 | | -0.025 ± 0.005 | | <0.0001 |
| Milk (g/day) | -0.002 ± 0.002 | | 0.31 | | -0.001 ± 0.0004 | | 0.17 | | -0.003 ± 0.002 | | 0.04 | -0.0005 ± 0.001 | | 0.63 | 0.003 ± 0.001 | | 0.04 | | -0.025 ± 0.006 | | <0.0001 |
| Liquid yogurt (g/day) | -0.011 ± 0.009 | | 0.21 | | -0.002 ± 0.002 | | 0.29 | | -0.005 ± 0.010 | | 0.62 | 0.002 ± 0.007 | | 0.84 | 0.005 ± 0.009 | | 0.59 | | 0.088 ± 0.049 | | 0.08 |
| Semisolid yogurt (g/day) | -0.003 ± 0.004 | | 0.47 | | -0.002 ± 0.001 | | 0.02 | | -0.005 ± 0.003 | | 0.08 | -0.003 ± 0.002 | | 0.22 | 0.004 ± 0.003 | | 0.22 | | -0.038 ± 0.014 | | 0.01 |
| Ice cream (g/day) | -0.002 ± 0.005 | | 0.72 | | -0.0001 ± 0.002 | | 0.97 | | -0.002 ± 0.005 | | 0.70 | -0.009 ± 0.004 | | 0.02 | -0.0004 ± 0.005 | | 0.93 | | -0.015 ± 0.023 | | 0.52 |
| Cheese (g/day) | -0.014 ± 0.015 | | 0.35 | | -0.010 ± 0.005 | | 0.07 | | -0.007 ± 0.017 | | 0.67 | 0.010 ± 0.013 | | 0.44 | 0.074 ± 0.019 | | 0.0001 | | -0.057 ± 0.065 | | 0.38 |
| Cream (g/day) | 0.005 ± 0.016 | | 0.75 | | 0.009 ± 0.006 | | 0.12 | | -0.018 ± 0.015 | | 0.22 | 0.004 ± 0.011 | | 0.69 | -0.013 ± 0.023 | | 0.57 | | -0.018 ± 0.069 | | 0.80 |
| One serving size of dairy products was defined as 200g for milk, 150 g for liquid yogurt, 100 g for semisolid yogurt, ice cream, and cream and 20 g for cheese. Dairy product intake was classified “0 serving size” for 0 intake, “0–1 serving size” for exceed 0 g and intake of less than one serving size, and “≥1 serving size” for intake of more than one serving size. | | | | | | | | | | | | | | | | | | | | | |
| ^1^Adjusted for age, BMI, education level, occupation, marital status, household income, household type, region, drinking, smoking, total energy intake, and physical activity. | | | | | | | | | | | | | | | | | | | | | |
| Multivariable logistic regression and linear regression were used to estimate the association between dairy products intake and components of metabolic syndrome. | | | | | | | | | | | | | | | | | | | | | |
| Abbreviations: NAFLD, non-alcoholic fatty liver disease; AOR, adjusted odds ratio; CI, confidence interval; HDL, high density lipoprotein; SE, standard error. | | | | | | | | | | | | | | | | | | | | | |

| **Table 5. Adjusted odds ratio (AOR) and 95% confidence intervals (CIs) for non-alcoholic fatty liver disease (NAFLD) according to components of metabolic syndrome** | | | | | |
| --- | --- | --- | --- | --- | --- |
| **Men (n=5,492)** | **AOR**^1^ **(95% CI)** | ***p*-value** | **Women (n = 7,238)** | **AOR (95% CI)** | ***p*-value** |
| **Hyperglycemia (mg/dL)** | | |  |  | |
| < 100 (n=2,908) | 1.00 (Ref.) | | < 100 (n=4,721) | 1.00 (Ref.) | |
| ≥ 100 (n=2,584) | 1.99 (1.60–2.48) | <0.0001 | ≥ 100 (n=2,562) | 2.82 (2.26–3.52) | <0.0001 |
| **Abdominal obesity (cm)** | | |  |  |  |
| < 90 (n=3,213) | 1.00 (Ref.) | | < 80 (n=3,576) | 1.00 (Ref.) | |
| ≥ 90 (n=2,279) | 1.90 (1.43–2.52) | <0.0001 | ≥ 80 (n=3,707) | 1.47 (1.50–2.06) | 0.02 |
| **Elevated blood pressure (mmHg)** | | |  |  | |
| SBP <130 and DBP <85 (n=2,920) | 1.00 (Ref.) | | SBP <130 and DBP <85 (n=4,484) | 1.00 (Ref.) | |
| SBP ≥130 or DBP ≥85 (n=2,572) | 1.36 (1.11–1.68) | 0.0039 | SBP ≥130 or DBP ≥85 (n=2,799) | 1.68 (1.32–2.14) | <0.0001 |
| **Low HDL-cholesterol (mg/dL)** | | |  |  | |
| > 40 (n=4,199) | 1.00 (Ref.) | | > 50 (n=4,783) | 1.00 (Ref.) | |
| ≤ 40 (n=1,293) | 1.64 (1.32–2.04) | <0.0001 | ≤ 50 (n=2,500) | 1.97 (1.61–2.41) | <0.0001 |
| **Elevated triglycerides (mg/dL)** | | |  |  | |
| < 150 (n=3,555) | 1.00 (Ref.) | | < 150 (n=5.839) | 1.00 (Ref.) | |
| ≥ 150 (n=1,973) | 1.97 (1.61–2.42) | <0.0001 | ≥ 150 (n=1,444) | 2.19 (1.75–2.73) | <0.0001 |
| ^1^Adjusted for age, BMI, education level, occupation, marital status, household income, household type, region, drinking, smoking, total energy intake, and physical activity. | | | | | |
| Multivariable logistic regression was used to estimate the association between NAFLD and components of metabolic syndrome. | | | | | |
| Abbreviations: NAFLD, non-alcoholic fatty liver disease; AOR, adjusted odds ratio; CI, confidence interval; HDL, high density lipoprotein. | | | | | |

5) Add Antioxidants such as vitamin E, ,Zn, Se, Mg, and amount of different kinds of fatty acids and amino acids to table 2.

***Response:*** Thank you very much for your suggestion.

We conducted additional analyses on antioxidant nutrients, including vitamin A, vitamin E, vitamin C, zinc, and magnesium, as well as fatty acids, including SFA, MUFA, and PUFA. Other nutrients could not be included as they were not assessed in the KNHANES survey.

It now reads “In men, individuals with NAFLD had significantly higher intakes of saturated fatty acids, monounsaturated fatty acids, and total fat compared to the normal group (p = 0.04, p = 0.02, p = 0.02, respectively), whereas polyunsaturated fatty acid intake was significantly lower in the NAFLD group (p = 0.04). Sodium intake was also significantly higher in men with NAFLD (p = 0.03). In women, individuals with NAFLD had significantly lower intakes of saturated fatty acids, monounsaturated fatty acids, polyunsaturated fatty acids, and total fat compared to the normal group (p = 0.008, p = 0.01, p = 0.003, p = 0.003, respectively). Additionally, vitamin E and calcium intake were significantly lower in women with NAFLD (p = 0.03, p = 0.04, respectively).”

| **Table 2. Nutrient intake of the participants by the status of non-alcoholic fatty liver disease status (NAFLD)** | | | | | | |
| --- | --- | --- | --- | --- | --- | --- |
| **Variables** | **Men (n=5,492)** | | ***p*-value** | **Women (n=7,283)** | | ***p*-value** |
|  | **NAFLD (HSI>36)** | **Normal** |  | **NAFLD (HSI>36)** | **Normal** |  |
| Total energy intake (kcal) | 2,081.95 ± 22.17 | 2,031.75 ± 15.00 | 0.054 | 1,561.12 ± 21.54 | 1,575.68 ± 10.38 | 0.53 |
| % energy of carbohydrate | 59.48 ± 0.36 | 60.58 ± 0.24 | 0.01 | 62.92 ± 0.40 | 61.24 ± 0.20 | 0.0001 |
| % energy of protein | 16.28 ± 0.16 | 16.16 ± 0.09 | 0.47 | 15.30 ± 0.16 | 15.32 ± 0.08 | 0.90 |
| % energy of fat | 24.23 ± 0.31 | 23.27 ± 0.20 | 0.01 | 21.77 ± 0.31 | 23.43 ± 0.17 | <0.0001 |
| Carbohydrate (g/day) | 304.25 ± 3.22 | 301.34 ± 2.14 | 0.44 | 241.66 ± 3.12 | 237.49 ± 1.59 | 0.23 |
| Protein (g/day) | 84.92 ± 1.21 | 82.43 ± 0.80 | 0.08 | 59.74 ± 1.13 | 60.39 ± 0.50 | 0.58 |
| Fat (g/day) | 58.37 ± 1.18 | 55.18 ± 0.75 | 0.02 | 39.50 ± 0.97 | 42.69 ± 0.48 | 0.003 |
| Dietary fiber (g/day) | 27.38 ± 0.37 | 28.18 ± 0.26 | 0.07 | 23.89 ± 0.40 | 23.24 ± 0.20 | 0.14 |
| Vitamin A (μg RAE/day) | 423.66 ± 14.24 | 414.88 ± 8.65 | 0.60 | 358.73 ± 9.90 | 379.58 ± 5.89 | 0.06 |
| Vitamin E (mg α-TE/day) | 7.90 ± 0.12 | 7.77 ± 0.08 | 0.35 | 6.02 ± 0.10 | 6.28 ± 0.06 | 0.03 |
| Vitamin C (mg/day) | 74.69 ± 6.02 | 65.32 ± 1.69 | 0.12 | 60.55 ± 2.14 | 66.24 ± 1.29 | 0.02 |
| β-Carotene (μg/day) | 2,973.29 ± 77.17 | 3,093.81 ± 72.35 | 0.26 | 2,732.49 ± 85.61 | 2,730.28 ± 46.22 | 0.98 |
| Magnesium (mg/day) | 331.53 ± 4.02 | 339.58 ± 2.65 | 0.10 | 270.33 ± 4.19 | 269.89 ± 1.99 | 0.92 |
| Saturated fatty acid (g/day) | 18.43 ± 0.41 | 17.48 ± 0.26 | 0.04 | 12.54 ± 0.36 | 13.69 ± 0.18 | 0.003 |
| Monounsaturated fatty acid (g/day) | 19.46 ± 0.47 | 18.18 ± 0.30 | 0.02 | 12.69 ± 0.35 | 13.65 ± 0.17 | 0.01 |
| Polyunsaturated fatty acids (g/day) | 14.62 ± 0.31 | 13.89 ± 0.19 | 0.04 | 10.05 ± 0.24 | 10.84 ± 0.13 | 0.003 |
| Zinc (mg/day) | 11.76 ± 0.16 | 11.72 ± 0.11 | 0.85 | 8.70 ± 0.14 | 8.71 ± 0.06 | 0.93 |
| Calcium (mg/day) | 532.29 ± 8.50 | 534.26 ± 5.50 | 0.84 | 441.22 ± 8.76 | 460.37 ± 4.36 | 0.04 |
| Phosphorus (mg/day) | 1,196.37 ± 13.75 | 1,198.31 ± 36.49 | 0.91 | 915.38 ± 14.44 | 931.78 ± 6.67 | 0.27 |
| Sodium (mg/day) | 3,958.56 ± 61.80 | 3,803.15 ± 36.49 | 0.03 | 2,772.08 ± .53.79 | 2,726.20 ± 25.46 | 0.44 |
| Potassium (mg/day) | 2,945.66 ± 34.51 | 2,999.71 ± 25.77 | 0.21 | 2,492.41 ± 40.64 | 2,478.52 ± 19.02 | 0.74 |
| Continuous variables are represented as mean ± standard error (SE). | | | | | | |
| The *p-*values were estimated using the t-test for continuous variables. | | | | | | |
| Abbreviations: NAFLD, non-alcoholic fatty liver disease; HSI, hepatic steatosis index; | | | | | | |

6) Please mention others research with more details for comparison their results with Your study results in the discussion.

***Response:*** The authors appreciate the reviewer’s recommendation. We added new contents and references.

“In addition to calcium's role in lipid metabolism, dairy components such as inulin-type fructans and whey protein have been suggested to modulate gut microbiota, potentially contributing to the inhibition of NAFLD progression through the gut-liver axis [21].”

“Consumption of more than 200 g of milk per day was related to a 12% decline in the abdominal obesity risk (95% CI: 0.79–0.97), and consuming more than 100 g of yogurt was related to a 16% reduced hyperglycemia risk (95% CI: 0.70–0.98) [23].”

Particularly, low-fat dairy products intake has been shown to have a positive effect on blood pressure regulation in elderly, and dairy products consumption has also been associated with lower blood pressure in children and adolescents [32, 33]. Furthermore, the consumption of low-fat dairy products has been reported to be more effective in lowering blood pressure, while full-fat dairy products have shown little or no effect [34].

“As indicated by prior research, men have been shown to have a higher tendency for visceral fat accumulation, which is associated with elevated blood pressure. In contrast, women primarily accumulate subcutaneous fat, which is less metabolically active. Consequently, men generally accumulate more visceral fat than women [41-43].”

References:

Reimer RA, Willis HJ, Tunnicliffe JM, Park H, Madsen KL, Soto‐Vaca A. Inulin‐type fructans and whey protein both modulate appetite but only fructans alter gut microbiota in adults with overweight/obesity: a randomized controlled trial. Mol Nutr Food Res. 2017;61(11):1700484.

Lee M, Lee H, Kim J. Dairy food consumption is associated with a lower risk of the metabolic syndrome and its components: a systematic review and meta-analysis. Br J Nutr. 2018;120(4):373-84. Epub 20180606. doi: 10.1017/s0007114518001460. PubMed PMID: 29871703.

Lana A, Banegas JR, Guallar-Castillón P, Rodríguez-Artalejo F, Lopez-Garcia E. Association of dairy consumption and 24-hour blood pressure in older adults with hypertension. Am J Med. 2018;131(10):1238-49.

Yuan WL, Kakinami L, Gray-Donald K, Czernichow S, Lambert M, Paradis G. Influence of dairy product consumption on children's blood pressure: results from the QUALITY cohort. JAND. 2013;113(7):936-41.

Alonso A, Beunza JJ, Delgado-Rodríguez M, Martínez JA, Martínez-González MA. Low-fat dairy consumption and reduced risk of hypertension: the Seguimiento Universidad de Navarra (SUN) cohort. Am J Clin. 2005;82(5):972-9.

Bredella MA. Sex Differences in Body Composition. Adv Exp Med Biol. 2017;1043:9-27.

Van Pelt RE, Jankowski CM, Gozansky WS, Wolfe P, Schwartz RS, Kohrt WM. Sex differences in the association of thigh fat and metabolic risk in older adults. Obesity. 2011;19(2):422-8.

Matsushita Y, Nakagawa T, Yamamoto S, Takahashi Y, Yokoyama T, Noda M, et al. Associations of visceral and subcutaneous fat areas with the prevalence of metabolic risk factor clustering in 6,292 Japanese individuals: the Hitachi Health Study. Diabetes Care. 2010;33(9):2117-9.

Once again, the authors truly appreciate all the constructive comments and suggestions from the reviewer.

**Response letter to Reviewer 2**

1) Author should discuss their findings rigorously to explain the reason behind the findings.

***Response:*** We appreciate your logical suggestions. The authors should rigorously discuss their findings to explain the biological mechanisms underlying the mediating effects of metabolic syndrome components between dairy product intake and NAFLD, and provide scientific evidence for the gender differences observed in these relationships as written below.

“In addition to calcium's role in lipid metabolism, dairy components such as inulin-type fructans and whey protein have been suggested to modulate gut microbiota, potentially contributing to the inhibition of NAFLD progression through the gut-liver axis [22].”

“Consumption of more than 200 g of milk per day was related to a 12% decline in the abdominal obesity risk (95% CI: 0.79–0.97), and consuming more than 100 g of yogurt was related to a 16% reduced hyperglycemia risk (95% CI: 0.70–0.98) [23].”

Particularly, low-fat dairy products intake has been shown to have a positive effect on blood pressure regulation in elderly, and dairy products consumption has also been associated with lower blood pressure in children and adolescents [32, 33]. Furthermore, the consumption of low-fat dairy products has been reported to be more effective in lowering blood pressure, while full-fat dairy products have shown little or no effect [34].

“As indicated by prior research, men have been shown to have a higher tendency for visceral fat accumulation, which is associated with elevated blood pressure. In contrast, women primarily accumulate subcutaneous fat, which is less metabolically active. Consequently, men generally accumulate more visceral fat than women [41-43].”

**References:**

Reimer RA, Willis HJ, Tunnicliffe JM, Park H, Madsen KL, Soto‐Vaca A. Inulin‐type fructans and whey protein both modulate appetite but only fructans alter gut microbiota in adults with overweight/obesity: a randomized controlled trial. Mol Nutr Food Res. 2017;61(11):1700484.

Lee M, Lee H, Kim J. Dairy food consumption is associated with a lower risk of the metabolic syndrome and its components: a systematic review and meta-analysis. Br J Nutr. 2018;120(4):373-84. Epub 20180606. doi: 10.1017/s0007114518001460. PubMed PMID: 29871703.

Lana A, Banegas JR, Guallar-Castillón P, Rodríguez-Artalejo F, Lopez-Garcia E. Association of dairy consumption and 24-hour blood pressure in older adults with hypertension. Am J Med. 2018;131(10):1238-49.

Yuan WL, Kakinami L, Gray-Donald K, Czernichow S, Lambert M, Paradis G. Influence of dairy product consumption on children's blood pressure: results from the QUALITY cohort. JAND. 2013;113(7):936-41.

Alonso A, Beunza JJ, Delgado-Rodríguez M, Martínez JA, Martínez-González MA. Low-fat dairy consumption and reduced risk of hypertension: the Seguimiento Universidad de Navarra (SUN) cohort. Am J Clin. 2005;82(5):972-9.

Bredella MA. Sex Differences in Body Composition. Adv Exp Med Biol. 2017;1043:9-27.

Van Pelt RE, Jankowski CM, Gozansky WS, Wolfe P, Schwartz RS, Kohrt WM. Sex differences in the association of thigh fat and metabolic risk in older adults. Obesity. 2011;19(2):422-8.

Matsushita Y, Nakagawa T, Yamamoto S, Takahashi Y, Yokoyama T, Noda M, et al. Associations of visceral and subcutaneous fat areas with the prevalence of metabolic risk factor clustering in 6,292 Japanese individuals: the Hitachi Health Study. Diabetes Care. 2010;33(9):2117-9.

Once again, the authors truly appreciate all the constructive comments and suggestions from the reviewer.
