# Supplementary material for: Examining the mediating effects of metabolic syndrome components on the relationship between dairy product consumption and nonalcoholic fatty liver disease in Korean adults
Source: PLoS One. 2026 Apr 10;21(4):e0346774. doi: 10.1371/journal.pone.0346774 (PMC13068242; doi:10.1371/journal.pone.0346774)
Supplement: Attachment — Submitted filename: Response to Reviewers.docx [file pone.0346774.s006.docx]

**Response letter to Reviewer 3**

1) In the Abstract and Results sections (page 13, line 31–32), it is stated that women consuming ≥1 serving of dairy had reduced odds of abdominal obesity (AOR: 0.84, 95% CI: 0.73–0.97). However, in Table 4 (page 23), the AOR for abdominal obesity is listed as 0.84 (95% CI: 0.73–0.97) under the "≥1 serving" group, but the footnote describes this CI as "1.50–2.06" (likely a typographical error).
***Response:*** The authors greatly appreciate the reviewer’s careful observation.

We have re-checked Table 4 as well as the Abstract and Results sections and confirmed that the AOR and 95% CI for abdominal obesity in women consuming ≥1 serving of dairy products are consistently reported as 0.84 (95% CI: 0.73–0.97). Therefore, no change was necessary to Table 4.

2) The manuscript states that "all variables used in the mediation analysis were continuous" (page 17, line 147). However, NAFLD (binary outcome) and MetS components (binary/categorical in regression models) were analyzed using logistic regression elsewhere.

***Response:*** Thank you very much for your suggestion.

In the mediation models, the independent variable (dairy product intake) and the mediator variables (MetS components) were entered as continuous measures. NAFLD, although classified as a binary outcome using a cut-off value of >36 from the Hepatic Steatosis Index (HSI) in logistic regression, was represented by its continuous HSI score in the mediation analysis. Using the continuous HSI score allowed us to capture the full spectrum of hepatic steatosis severity, preserve variability, and meet the linearity assumptions of the bootstrapping approach implemented in the SAS macro.

We have added the Methods section to clarify this point as follows: “*NAFLD, although classified as a binary outcome using a cut-off value of >36 on the HSI in logistic regression, was represented by its continuous HSI score in the mediation analysis. Using the continuous HSI score allowed us to capture the full spectrum of hepatic steatosis severity, preserve variability, and meet the linearity assumptions of the bootstrapping approach implemented in the SAS macro.*”

3) While covariates (age, BMI, lifestyle factors) were adjusted for in regression models, total energy intake was not included as a covariate in: Mediation analyses (page 17, lines 145–150); Associations between dairy intake and MetS components (Table 4).

***Response:*** Thank you for pointing this out.

We would like to clarify that total energy intake was included as a covariate in all regression models, including both the mediation analyses and the analyses of associations between dairy intake and MetS components. The footnotes of Table 4 and Figure 2 already indicate that total energy intake was adjusted for. However, it was inadvertently omitted from the covariate list in the Statistical Analyses section, which may have caused confusion. We have now revised this section to explicitly state “*... after controlling for covariates such as age, education level, income, employment status, marital status, household type, region, drinking and smoking status, total energy intake, physical activity, and BMI*” to avoid ambiguity.

4) The study aggregates all dairy types (milk, yogurt, cheese, etc.) but notes differential effects of low-fat vs. full-fat dairy on blood pressure (pages 28–29, 33). Despite this, stratified analyses by dairy type/fat content are limited.

***Response:*** We appreciate the reviewer’s comment regarding stratified analyses by dairy fat content.

We acknowledge that low-fat and full-fat dairy products may exert differential physiological effects. However, the KNHANES dataset does not provide detailed fat content information for all dairy subtypes, which limits our ability to perform stratified analyses by fat content. Nevertheless, as noted in the Discussion section, “*Some studies have reported that low-fat dairy consumption was inversely associated with blood pressure, whereas the association with full-fat dairy was inconsistent*”. While we were unable to perform separate analyses by fat content, by citing these studies, we acknowledge and contextualize potential variations in health effects according to dairy fat content within the interpretation of our findings.

Once again, the authors truly appreciate all the constructive comments and suggestions from the reviewer.
