# Supplementary material for: Examining the mediating effects of metabolic syndrome components on the relationship between dairy product consumption and nonalcoholic fatty liver disease in Korean adults
Source: PLoS One. 2026 Apr 10;21(4):e0346774. doi: 10.1371/journal.pone.0346774 (PMC13068242; doi:10.1371/journal.pone.0346774)
Supplement: Attachment — Submitted filename: Comments.docx [file pone.0346774.s005.docx]

1. In Table3, The analysis of the relationship between dairy product intake and NAFLD relies on a three-category exposure with the lowest category set as the reference. This approach implicitly assumes stepwise monotonic differences between categories and may obscure a non-linear dose–response pattern. It is possible that the association between dairy intake and NAFLD is not linear; therefore, using flexible methods such as restricted cubic splines or fractional polynomials to test for non-linearity in order to reduce the risk of misclassification arising from coarse categorization.
2. The mediation analysis examining the relationship between dairy product consumption and NAFLD should clarify how medication use was handled, particularly lipid-lowering agents.
3. The manuscript should more explicitly address how baseline and concurrent liver status—such as fibrosis stage and liver enzyme levels (e.g., ALT, AST, GGT, ALP)—was handled in the mediation analysis (Figure2), as these factors are closely related to both NAFLD development/progression and potential exposures/mediators. Without clear treatment of these variables, there is a risk of confounding
