# Supplementary material for: Examining the mediating effects of metabolic syndrome components on the relationship between dairy product consumption and nonalcoholic fatty liver disease in Korean adults
Source: PLoS One. 2026 Apr 10;21(4):e0346774. doi: 10.1371/journal.pone.0346774 (PMC13068242; doi:10.1371/journal.pone.0346774)
Supplement: Attachment — Submitted filename: Response to Reviewers_Final.docx [file pone.0346774.s007.docx]

**Response letter to Reviewer 5**

1) In Table3, the analysis of the relationship between dairy product intake and NAFLD relies on a three-category exposure with the lowest category set as the reference. This approach implicitly assumes stepwise monotonic differences between categories and may obscure a non-linear dose–response pattern. It is possible that the association between dairy intake and NAFLD is not linear; therefore, using flexible methods such as restricted cubic splines or fractional polynomials to test for non-linearity in order to reduce the risk of misclassification arising from coarse categorization.
   ***Response:*** The authors greatly appreciate the reviewer’s careful observation.

As recommended, we conducted an additional **restricted cubic spline (RCS)** analysis to examine the potential non-linear association between dairy product intake and NAFLD.

The spline model showed a **non-linear inverse trend,** with a gradual decrease in NAFLD risk at low levels of dairy product intake and a plateau thereafter. However, the overall pattern remained **consistent with the results** presented in Table 3, and the main findings and conclusions **were not materially changed.**

Given that the spline analysis did not alter the substantive interpretation, we decided to **retain the simple model** in the main text for clarity and interpretability, while providing the RCS results and figure in the Supplementary Materials for reference.

This approach allows us to fully address the reviewer’s comment, and the results are visually presented in the Figure 1 below.

Figure 1. Restricted Cubic Spline of Dairy product intake and non-alcoholic fatty liver disease by sex. (Blue indicates men, and red indicates women.)

2) The mediation analysis examining the relationship between dairy product consumption and NAFLD should clarify how medication use was handled, particularly lipid-lowering agents.

***Response:*** Thank you very much for your suggestion.

The Korea National Health and Nutrition Examination Survey (KNHANES 2019–2021) includes a variable indicating whether participants are taking medication for dyslipidemia. However, the dataset does not provide information on which lipid component (e.g., LDL, TG, HDL) the medication specifically targets. Additionally, details such as medication type, dosage, and combination therapy are not collected, making it difficult to accurately distinguish or interpret the effects of such medications. Due to these limitations, this variable was excluded from the mediation model, and the potential for residual confounding has been acknowledged as a limitation in the Discussion section of the manuscript.

It now reads *“In addition, although the KNHANES dataset includes a variable indicating whether participants were taking medication for dyslipidemia, it lacks information on medication type, dosage, therapeutic target (e.g., LDL, HDL, TG), and combination therapy, so it could not be considered in the analysis.”*.

3) The manuscript should more explicitly address how baseline and concurrent liver status—such as fibrosis stage and liver enzyme levels (e.g., ALT, AST, GGT, ALP)—was handled in the mediation analysis (Figure2), as these factors are closely related to both NAFLD development/progression and potential exposures/mediators. Without clear treatment of these variables, there is a risk of confounding

***Response:*** Thank you for pointing this out.

In this study, non-alcoholic fatty liver disease (NAFLD) was defined using the Hepatic Steatosis Index (HSI), which incorporates the ALT/AST ratio, body mass index (BMI), sex, and diabetes status. Because ALT and AST are integral components of the HSI formula, they were not included as separate covariates in the mediation analysis.

Unfortunately, the Korea National Health and Nutrition Examination Survey (KNHANES 2019–2021) dataset used in this study does not contain data on GGT, ALP, or fibrosis-related indices such as FIB-4 or NFS. Therefore, these variables could not be included in our analyses.

It now reads *“Furthermore, the dataset does not provide information on liver fibrosis stage or liver enzyme levels such as GGT or ALP, which may also influence NAFLD risk.”*.

Once again, the authors truly appreciate all the constructive comments and suggestions from the reviewer.
