# Supplementary material for: Examining the mediating effects of metabolic syndrome components on the relationship between dairy product consumption and nonalcoholic fatty liver disease in Korean adults
Source: PLoS One. 2026 Apr 10;21(4):e0346774. doi: 10.1371/journal.pone.0346774 (PMC13068242; doi:10.1371/journal.pone.0346774)
Supplement: Attachment — Submitted filename: Response to Reviewers_DS.docx [file pone.0346774.s008.docx]

**Title: Examining the mediating effects of metabolic syndrome components on the relationship between dairy product consumption and nonalcoholic fatty liver disease in Korean adults**

**Response letter to Reviewer 6**

We highly appreciate your insightful and helpful comments. Through your comments, we could improve the overall quality of our manuscript. However, prior to the final submission, we carefully reanalyzed the data to ensure reproducibility and identified several minor errors, which have now been corrected. Accordingly, we thoroughly reviewed the manuscript to reflect these changes and to incorporate the reviewers’ suggestions as fully as possible. All page and line numbers refer to the revised manuscript file without track changes.

**Comment 1)** Please consider adding sensitivity analyses that use alternative NAFLD indices where possible, or analyses stratified by key variables like age and BMI.
***Response:*** We appreciate the reviewer’s constructive suggestion.

In response, we conducted sensitivity analyses stratified by age and BMI to evaluate the robustness of the mediation effects. The results were added as Supplementary Tables.

Across age and BMI strata, the overall pattern of mediation remained consistent with the main findings, and the number of statistically significant natural indirect effects (NIEs) was consistently greater among women than among men, supporting the robustness of the observed sex-specific associations.

**“**Sensitivity analyses using age- and BMI-stratified mediation models were conducted, with a greater number of NIEs observed among women than among men across strata (Supplementary tables 1 and 2).”

**Supplementary table 1. Mediation effects of metabolic syndrome components on the association between dairy product consumption and NAFLD, stratified by age**

|  | Age (years) | Dairy → mediator (β₁) | Mediator → NAFLD (β₂) | NIE | NDE | Total effect | Proportion mediated |
| --- | --- | --- | --- | --- | --- | --- | --- |
| *Men* |  |  |  |  |  |  |  |
| Fasting blood glucose | 19-39 | 0.00159 | **0.01611*** | 0.00003 | 0.00007 | 0.00009 | 0.27073 |
|  | 40-64 | 0.00013 | **0.01991*** | 0.00000 | 0.00002 | 0.00002 | 0.12232 |
|  | ≥65 | -0.00976 | **0.02854*** | -0.00028 | 0.00039 | 0.00011 | NA |
| Waist circumference | 19-39 | -0.00110 | **0.18011*** | -0.00020 | 0.00029 | 0.00009 | NA |
|  | 40-64 | -0.00102 | **0.08945*** | -0.00009 | 0.00011 | 0.00002 | NA |
|  | ≥65 | -0.00158 | **0.03154*** | -0.00005 | 0.00016 | 0.00011 | -0.43644 |
| SBP | 19-39 | -0.00119 | **0.02136*** | -0.00003 | 0.00012 | 0.00009 | -0.26862 |
|  | 40-64 | **-0.00470*** | 0.00125 | -0.00001 | 0.00003 | 0.00002 | -0.27693 |
|  | ≥65 | -0.00633 | 0.00264 | -0.00002 | 0.00013 | 0.00011 | -0.14627 |
| DBP | 19-39 | -0.00253 | **0.04341*** | -0.00011 | 0.00020 | 0.00009 | NA |
|  | 40-64 | -0.00135 | **0.01393*** | -0.00002 | 0.00004 | 0.00002 | -0.88872 |
|  | ≥65 | -0.00082 | 0.01072 | -0.00001 | 0.00012 | 0.00011 | -0.07667 |
| HDL-cholesterol | 19-39 | -0.00118 | **-0.04987*** | 0.00006 | 0.00004 | 0.00009 | 0.62197 |
|  | 40-64 | 0.00291 | **-0.03345*** | -0.00010 | 0.00012 | 0.00002 | NA |
|  | ≥65 | 0.00168 | **-0.01422*** | -0.00002 | 0.00014 | 0.00011 | -0.20847 |
| Triglycerides | 19-39 | -0.02013 | **0.00542*** | -0.00011 | 0.00020 | 0.00009 | NA |
|  | 40-64 | **-0.05837*** | **0.00172*** | **-0.00010*** | 0.00012 | 0.00002 | NA |
|  | ≥65 | -0.03340 | **0.00283*** | -0.00009 | 0.00021 | 0.00011 | -0.82574 |
| *Women* |  |  |  |  |  |  |  |
| Fasting blood glucose | 19-39 | -0.00360 | **0.03001*** | -0.00011 | -0.00050 | -0.00061 | 0.17825 |
|  | 40-64 | -0.00307 | **0.03069*** | -0.00009 | 0.00017 | 0.00008 | NA |
|  | ≥65 | -0.00241 | **0.03168*** | -0.00008 | 0.00008 | 0.00001 | NA |
| Waist circumference | 19-39 | -0.00025 | **0.06066*** | -0.00002 | -0.00059 | -0.00061 | 0.02530 |
|  | 40-64 | **-0.00136*** | **0.07076*** | **-0.00010*** | 0.00018 | 0.00008 | NA |
|  | ≥65 | **-0.00218*** | **0.06339*** | **-0.00014*** | 0.00014 | 0.00001 | NA |
| SBP | 19-39 | **-0.00344*** | 0.01006 | -0.00003 | -0.00057 | -0.00061 | 0.05715 |
|  | 40-64 | **-0.00571*** | **0.00931*** | **-0.00005*** | 0.00013 | 0.00008 | -0.66901 |
|  | ≥65 | 0.00047 | 0.00254 | 0.00000 | 0.00001 | 0.00001 | 0.19119 |
| DBP | 19-39 | -0.00089 | 0.00621 | -0.00001 | -0.00060 | -0.00061 | 0.00911 |
|  | 40-64 | -0.00196 | **0.01120*** | -0.00002 | 0.00010 | 0.00008 | -0.27631 |
|  | ≥65 | -0.00065 | 0.00208 | 0.00000 | 0.00001 | 0.00001 | -0.21897 |
| HDL-cholesterol | 19-39 | 0.00154 | **-0.02501*** | -0.00004 | -0.00057 | -0.00061 | 0.06366 |
|  | 40-64 | **0.00437*** | **-0.02412*** | **-0.00011*** | 0.00018 | 0.00008 | NA |
|  | ≥65 | 0.00390 | -0.00540 | -0.00002 | 0.00003 | 0.00001 | NA |
| Triglycerides | 19-39 | -0.01372 | **0.00807*** | -0.00011 | -0.00050 | -0.00061 | 0.18257 |
|  | 40-64 | **-0.04494*** | **0.00506*** | **-0.00023*** | 0.00031 | 0.00008 | NA |
|  | ≥65 | -0.01309 | **0.00370*** | -0.00005 | 0.00005 | 0.00001 | NA |

Statistically significant values are indicated in bold **(**p* < 0.05**).

β₁ represents the regression coefficient for the association between dairy product consumption and the metabolic syndrome component.

β₂ represents the regression coefficient for the association between the metabolic syndrome component and NAFLD.

The proportion mediated was calculated as NIE divided by the total effect and is reported as NA when the value exceeded 1.

All models were adjusted for BMI, education level, occupation, marital status, household income, household type, region, drinking, smoking, total energy intake, and physical activity.

NAFLD, non-alcoholic fatty liver disease; NIE, natural indirect effect; NDE, natural direct effect; SBP, systolic blood pressure; DBP, diastolic blood pressure; HDL, high-density lipoprotein.

**Supplementary table 2. Mediation effects of metabolic syndrome components on the association between dairy product consumption and NAFLD, stratified by BMI**

|  | BMI (kg/m^2^) | Dairy → mediator (β₁) | Mediator → NAFLD (β₂) | NIE | NDE | Total effect | Proportion mediated |
| --- | --- | --- | --- | --- | --- | --- | --- |
| *Men* |  |  |  |  |  |  |  |
| Fasting blood glucose | <18.5 | -0.00534 | 0.02086 | -0.00011 | -0.00050 | -0.00061 | 0.18230 |
|  | 18.5-22.9 | -0.00525 | **0.03651*** | -0.00019 | 0.00041 | 0.00022 | -0.87601 |
|  | 23.0-24.9 | 0.00132 | **0.02393*** | 0.00003 | -0.00017 | -0.00014 | -0.22524 |
|  | ≥25 | -0.00249 | **0.03617*** | -0.00009 | -0.00077 | -0.00086 | 0.10501 |
| Waist circumference | <18.5 | -0.00208 | **0.12025*** | -0.00025 | -0.00036 | -0.00061 | 0.40907 |
|  | 18.5-22.9 | -0.00144 | **0.26849*** | -0.00039 | 0.00060 | 0.00022 | NA |
|  | 23.0-24.9 | -0.00135 | **0.18938*** | -0.00026 | 0.00012 | -0.00014 | NA |
|  | ≥25 | **-0.00264*** | **0.39727*** | **-0.00105*** | 0.00019 | -0.00086 | NA |
| SBP | <18.5 | -0.00294 | 0.02099 | -0.00006 | -0.00055 | -0.00061 | 0.10116 |
|  | 18.5-22.9 | -0.00373 | 0.00904 | -0.00003 | 0.00025 | 0.00022 | -0.15414 |
|  | 23.0-24.9 | -0.00489 | 0.00910 | -0.00004 | -0.00010 | -0.00014 | 0.31851 |
|  | ≥25 | **-0.00393*** | **0.04209*** | -0.00017 | -0.00069 | -0.00086 | 0.19288 |
| DBP | <18.5 | -0.00243 | 0.03368 | -0.00008 | -0.00053 | -0.00061 | 0.13389 |
|  | 18.5-22.9 | -0.00171 | **0.01659*** | -0.00003 | 0.00025 | 0.00022 | -0.12962 |
|  | 23.0-24.9 | **-0.00418*** | **0.02939*** | -0.00012 | -0.00002 | -0.00014 | 0.87830 |
|  | ≥25 | -0.00225 | **0.06730*** | -0.00015 | -0.00071 | -0.00086 | 0.17686 |
| HDL-cholesterol | <18.5 | -0.00435 | -0.00171 | 0.00001 | -0.00062 | -0.00061 | -0.01221 |
|  | 18.5-22.9 | 0.00118 | **-0.04893*** | -0.00006 | 0.00028 | 0.00022 | -0.26350 |
|  | 23.0-24.9 | 0.00246 | **-0.02254*** | -0.00006 | -0.00008 | -0.00014 | 0.39699 |
|  | ≥25 | 0.00112 | **-0.08353*** | -0.00009 | -0.00076 | -0.00086 | 0.10867 |
| Triglycerides | <18.5 | -0.01174 | 0.00136 | -0.00002 | -0.00059 | -0.00061 | 0.02612 |
|  | 18.5-22.9 | -0.03411 | **0.00354*** | -0.00012 | 0.00034 | 0.00022 | -0.55238 |
|  | 23.0-24.9 | -0.02678 | **0.00257*** | -0.00007 | -0.00007 | -0.00014 | 0.49329 |
|  | ≥25 | **-0.06365*** | **0.00417*** | **-0.00027*** | -0.00059 | -0.00086 | 0.30988 |
| *Women* |  |  |  |  |  |  |  |
| Fasting blood glucose | <18.5 | -0.00009 | **0.03867*** | 0.00000 | 0.00144 | 0.00144 | -0.00231 |
|  | 18.5-22.9 | -0.00352 | **0.03619*** | -0.00013 | -0.00007 | -0.00020 | 0.63085 |
|  | 23.0-24.9 | -0.00699 | **0.03674*** | -0.00026 | 0.00010 | -0.00015 | NA |
|  | ≥25 | 0.00048 | **0.04421*** | 0.00002 | -0.00067 | -0.00065 | -0.03253 |
| Waist circumference | <18.5 | 0.00002 | **0.11913*** | 0.00000 | 0.00144 | 0.00144 | 0.00198 |
|  | 18.5-22.9 | **-0.00141*** | **0.22519*** | **-0.00032*** | 0.00012 | -0.00020 | NA |
|  | 23.0-24.9 | **-0.00187*** | **0.10469*** | **-0.00020*** | 0.00004 | -0.00015 | NA |
|  | ≥25 | -0.00163 | **0.40088*** | -0.00066 | 0.00001 | -0.00065 | NA |
| SBP | <18.5 | 0.00008 | -0.01069 | 0.00000 | 0.00144 | 0.00144 | -0.00062 |
|  | 18.5-22.9 | **-0.00445*** | **0.00781*** | -0.00003 | -0.00017 | -0.00020 | 0.17191 |
|  | 23.0-24.9 | -0.00004 | **0.01190*** | 0.00000 | -0.00015 | -0.00015 | 0.00317 |
|  | ≥25 | -0.00471 | **0.04477*** | -0.00021 | -0.00044 | -0.00065 | 0.32650 |
| DBP | <18.5 | 0.00040 | -0.01643 | -0.00001 | 0.00145 | 0.00144 | -0.00460 |
|  | 18.5-22.9 | **-0.00236*** | 0.00833 | -0.00002 | -0.00018 | -0.00020 | 0.09727 |
|  | 23.0-24.9 | 0.00077 | **0.02216*** | 0.00002 | -0.00017 | -0.00015 | -0.11191 |
|  | ≥25 | -0.00122 | **0.07555*** | -0.00009 | -0.00055 | -0.00065 | 0.14266 |
| HDL-cholesterol | <18.5 | 0.00953 | -0.01071 | -0.00010 | 0.00154 | 0.00144 | -0.07086 |
|  | 18.5-22.9 | **0.00353*** | **-0.02847*** | **-0.00010*** | -0.00010 | -0.00020 | 0.49741 |
|  | 23.0-24.9 | -0.00035 | **-0.02511*** | 0.00001 | -0.00016 | -0.00015 | -0.05761 |
|  | ≥25 | **0.00511*** | **-0.05482*** | **-0.00028*** | -0.00037 | -0.00065 | 0.43360 |
| Triglycerides | <18.5 | **-0.03776*** | 0.00536 | -0.00020 | **0.00164*** | 0.00144 | -0.14069 |
|  | 18.5-22.9 | **-0.01567*** | **0.00715*** | **-0.00011*** | -0.00009 | -0.00020 | 0.55494 |
|  | 23.0-24.9 | -0.02664 | **0.00633*** | -0.00017 | 0.00002 | -0.00015 | NA |
|  | ≥25 | **-0.05846*** | **0.00951*** | **-0.00056*** | -0.00009 | -0.00065 | 0.86062 |

Statistically significant values are indicated in bold **(**p* < 0.05**).

β₁ represents the regression coefficient for the association between dairy product consumption and the metabolic syndrome component.

β₂ represents the regression coefficient for the association between the metabolic syndrome component and NAFLD.

The proportion mediated was calculated as NIE divided by the total effect and is reported as NA when the value exceeded 1.

All models were adjusted for age, education level, occupation, marital status, household income, household type, region, drinking, smoking, total energy intake, and physical activity.

NAFLD, non-alcoholic fatty liver disease; NIE, natural indirect effect; NDE, natural direct effect; SBP, systolic blood pressure; DBP, diastolic blood pressure; HDL, high-density lipoprotein.

**Comment 2)** It would strengthen the paper to discuss the possible biological reasons for the sex differences observed—such as hormonal or genetic factors involved in metabolic syndrome.

***Response:*** We thank the reviewer for this helpful comment.

In response, we have expanded the Discussion to address the potential biological mechanisms underlying the observed sex differences. Specifically, we added a new paragraph describing sex-specific metabolic responses to dairy consumption, including differences in adipose tissue distribution, insulin sensitivity, and lipid metabolism. We also incorporated evidence from clinical trials demonstrating differential metabolic responses to dairy intake between men and women.

“*Sex-specific associations of dairy consumption with metabolic syndrome and NAFLD”*
“The present study demonstrated that the associations between dairy consumption, NAFLD, and MetS components vary by sex, with significant associations observed only among women. Similar sex-specific patterns have been reported in previous studies. A cohort study found that higher dairy consumption was more strongly associated with a lower prevalence of NAFLD in women [45], a finding that was supported by a meta-analysis of observational studies reporting consistent results [46]. Regarding MetS components, a 10-year cohort study from the Korean Genome and Epidemiology Study (KoGES) showed that dairy consumption was associated with a reduced risk of low HDL-cholesterol among women [47], and a cross-sectional study using KNHANES observed a lower MetS prevalence among Korean women consuming $\geq$1 serving/day of dairy products [48]. Moreover, MetS and its individual components have been shown to be more strongly associated with NAFLD in women than in men [49].

These sex differences may partly arise because the metabolic effects of diets and dietary constituents are expressed in a sex-specific manner and are highly dependent on disease context [50]. Clinical evidence further supports sex-specific metabolic responses to dairy consumption. In a 6-week randomized controlled trial (RCT), low-fat dairy intake differentially improved MetS parameters by sex, with a modest but significant reduction in fasting blood glucose in men and reductions in waist circumference, body weight, and BMI in women [51]. Another RCT demonstrated distinct postprandial lipoprotein responses to the dairy between sexes, with women exhibiting smaller increases in VLDL particles and greater increases in HDL particle concentrations than men [52]. Biological sex also exerts a strong influence on adipose tissue distribution and metabolic function. Women generally have greater amounts of subcutaneous adipose tissue than men [53], and although abdominal subcutaneous fat has been associated with adverse glucose and lipid profiles [54], subcutaneous fat may provide partial protection against metabolic dysfunction, thereby potentially reducing the risk of MetS in women [55]. In addition, insulin sensitivity differs by sex, with women typically exhibiting greater adipose tissue insulin sensitivity than men [56]. Consistently, dairy intake has been shown to inhibit intestinal fat absorption, improve lipid profiles, reduce blood pressure, and enhance glucose metabolism [57-59], thereby contributing to improvements in NAFLD. Sex hormones further modulate these processes, with estrogens promoting metabolically protective pathways and androgens supporting male-specific metabolic environments [60]. Collectively, these biological and clinical differences suggest that dairy exposure may engage distinct metabolic pathways in men and women, contributing to the observed sex-specific associations.”

**Comment 3)** Also, please expand on how the serving sizes used in your study relate to clinical practice, and discuss whether your findings can be applied to populations outside Korea, given the differences in dairy products across countries.

***Response:*** We are grateful for the reviewer’s thoughtful recommendation.

We have expanded the Discussion to address the clinical relevance of our serving size criteria and the generalizability of our findings to populations outside Korea.

The serving sizes used in this study (200 g for milk, 100–150 g for yogurt, and 20 g for cheese) are consistent with standard portions recommended in international dietary guidelines [26, 61, 62] and commonly used in clinical nutrition practice. These amounts represent realistic, achievable targets for dietary interventions. For instance, 200 g of milk is equivalent to a standard glass, making our findings directly translatable to practical dietary recommendations. Regarding generalizability, while we acknowledge that specific dairy product preferences vary across countries, our analytical approach enhances the external validity of our findings. Our primary mediation analyses focused on total dairy intake, which captures the overall contribution of dairy consumption regardless of specific products consumed. We also conducted sensitivity analyses examining individual dairy types (milk, yogurt, and cheese separately), allowing interpretation across diverse dietary contexts. However, we acknowledge that the relatively low cheese consumption in our Korean cohort may limit the precision of cheese-specific estimates when applied to populations with higher cheese intake patterns.

“The serving sizes used in this study (200 g for milk, 100–150 g for yogurt, and 20 g for cheese) are consistent with standard portions in international dietary guidelines [26, 61, 62] and represent realistic targets for clinical dietary counseling. Although dairy product preferences differ across countries, our focus on total dairy intake in the primary mediation analysis supports the broader applicability of our findings. Sensitivity analyses examining individual dairy types allow for interpretation across diverse dietary contexts, though the generalizability y of cheese-specific findings may be limited given the relatively low cheese consumption in our Korean cohort compared to Western populations.”

Once again, the authors truly appreciate all the constructive comments and suggestions from the reviewer.
